# Interplay between element-specific distortions and electrocatalytic oxygen evolution for cobalt–iron hydroxides†

**DOI:** 10.1039/d4sc01841a

**Published:** 2024-08-27

**Authors:** Elif Pınar Alsaç, Marlyn Boke, Justine R. Bissonnette, Rodney D. L. Smith

**Affiliations:** a Department of Chemistry, University of Waterloo, 200 University Avenue W. Waterloo Ontario N2L 3G1 Canada rodsmith@uwaterloo.ca; b Waterloo Institute for Nanotechnology, University of Waterloo, 200 University Avenue W. Waterloo Ontario N2L 3G1 Canada; c Waterloo Artificial Intelligence Institute, University of Waterloo, 200 University Avenue W. Waterloo Ontario N2L 3G1 Canada

## Abstract

A microscopic understanding of how Fe-doping of Co(OH)_2_ improves electrocatalytic oxygen evolution remains elusive. We study two Co_1−*x*_Fe_*x*_(OH)_2_ series that differ in fabrication protocol and find composition alone poorly correlates to catalyst performance. Structural descriptors extracted using X-ray diffraction, X-ray absorption spectroscopy, and Raman spectroscopy reveal element-specific distortions in Co_1−*x*_Fe_*x*_(OH)_2_. These structural descriptors are composition-dependent within individual sample series but inconsistent across fabrication protocols, revealing fabrication-dependence in catalyst microstructure. Correlations between structural parameters from different techniques show that Fe–O resists bond length changes, forcing distortion of Co environments. We find the difference in O–M–O bond angles between Co and Fe sites to correlate with electrocatalytic behavior across both sample series, which we attribute to asymmetric distortion of potential energy surfaces for the Co(iii) to Co(iv) oxidation. A Tafel slope consistent with a rate-limiting step without electron transfer emerges as the O–Co–O angle decreases, implying a distortion-induced transition in the rate-limiting step. The fabrication dependence of electronic and bonding structure in the catalysts should be considered in theoretical and high-throughput analyses of electrocatalyst materials.

## Abstract 2

The response of Co_1−*x*_Fe_*x*_(OH)_2_ electrocatalysts for the oxygen evolution reaction to changes in voltage correlates to element specific distortions. These angular distortions are caused by inequality between Fe–O and Co–O bond lengths.

## Graphical abstract

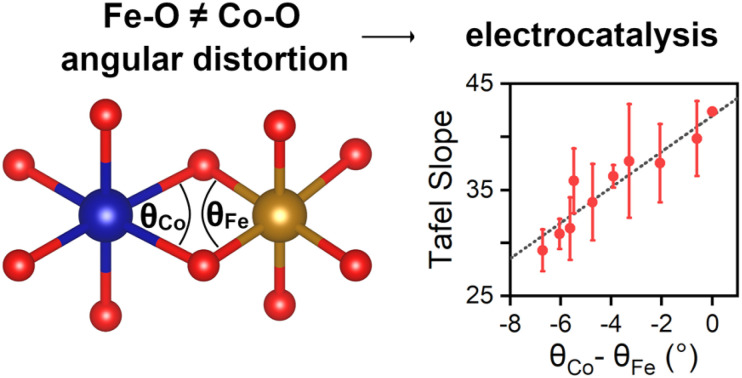

## Introduction

Electrocatalysts are being developed to enable sustainable fuel synthesis through various cathodic reactions, including hydrogen evolution, CO_2_ reduction, and nitrogen reduction. These reactions are reliant upon a source of electrons and protons, for which the oxygen evolution reaction (OER) may be the only option suitable for a global scale.^1,2^ Detailed microscopic understanding of electrocatalysts for OER – how specific structural features affect electrochemical behavior – will enable rational design of reaction systems as breakthroughs in cathodic electrocatalysts occur.

Structure–property analyses are a primary tool for establishing microscopic understanding of electrocatalysts,^3^ but dynamic changes of catalyst structure impose challenges.^4^ Crystalline materials such as perovskites and spinels are commonly analyzed due to ease of structural characterization and an amenability to compositional tuning. Analyses of these families have revealed relationships between electrocatalytic performance and structural descriptors such as d-orbital occupancies,^5^ bond covalency,^6–9^ twisting distortions between neighboring polyhedra,^10^ and local symmetry changes.^11^ Reports of electrocatalyst amorphization or catalyst restructuring,^12,13^ with some reports even purporting structural regeneration upon termination of experiments,^14,15^ present a problem. Structural disruption of crystalline materials often yields disordered materials with structural characteristics of a layered double (oxy)hydroxide (M(O)_2−*y*_(OH)_*y*_), a family which spans from metal hydroxides (M(OH)_2_) to metal oxyhydroxides (MOOH), as in the extensively studied CoPi catalyst.^16^

Disordered cobalt-based (oxy)hydroxide materials have been extensively studied, with X-ray absorption fine structure spectroscopy (XAFS) being a prominent tool for analysis.^17^ Blending Fe into the structure to form Co_1−*x*_Fe_*x*_(OH)_2_ is known to improve OER, but experiments have supported diverse proposals. Proposals include partial oxidation of Fe sites that promote OER through cobalt sites,^18^ or act directly as catalyst sites,^19^ and that multiple electroactive sites activate at different potentials.^20^ Our analysis of isostructural Ni_1−*x*_Fe_*x*_(OH)_2_ found localized geometric distortions that correlated with improved OER;^21–23^ the distortions were easier to study when increased crystallinity decreased the magnitude of strain.^24^ This finding motivated us to study Co_1−*x*_Fe_*x*_(OH)_2_, where the more comparable ionic radii should moderate strain.

Herein, we report a structural parameter that correlates to the catalytic OER responsiveness of Co_1−*x*_Fe_*x*_(OH)_2_ materials synthesized by different techniques. Two series of samples were synthesized with the same nominal composition using different protocols, with samples denoted W*x* when synthesized in water and FA*x* when in the presence of formamide (*x* from 0 to 0.3 in steps of 0.05). Electrochemical behavior of the series is qualitatively similar but lacking quantitative trends. Structure–property correlations based on X-ray diffraction (XRD), XAFS, Raman spectroscopy, and electrochemistry reveal differences in localized coordination environments for the Co and Fe ions. The difference in oxygen–metal–oxygen bond angles for Co and Fe is found to correlate with Tafel slopes for OER, which identifies a viable catalyst design strategy.

## Results

**Fig. 1:**
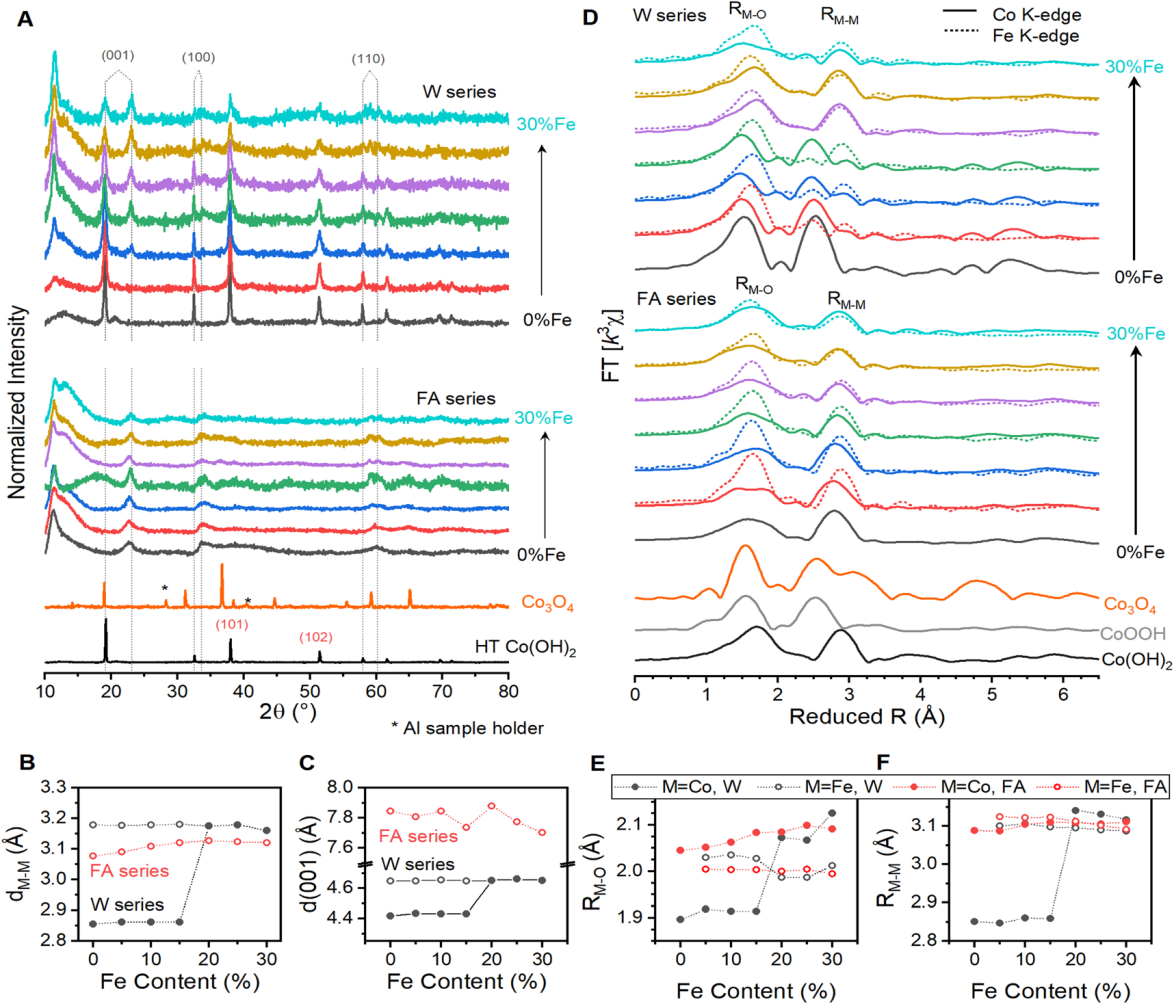
Structural analysis of the two series of Co_1−*x*_Fe_*x*_(OH)_2_ samples. (A) Powder X-ray diffraction patterns for W0 through W30 (top), FA0 through FA30 (bottom), with Co_3_O_4_ and highly crystalline β-Co(OH)_2_ synthesized for comparison. Effect of composition on (B) the distance between nearest transition metal neighbors as determined from the (100) Bragg peak, and (C) interlayer spacing. The data for the water series shows distances measured immediately after synthesis (hollow points) and after four weeks (solid points). (D) Fourier transformed EXAFS results for the water (top) and formamide (center) sample series, with hydrothermally synthesized Co(OH)_2_, an electrochemically oxidized form of it (CoOOH), and Co_3_O_4_ included for comparison (bottom). Composition dependent trends in (E) *R*_M–O_ and (F) *R*_M–M_ for the two series. Each composition series contains Fe contents of 0% (black), 5% (red), 10% (blue), 15% (green), 20% (purple), 25% (brown), and 30% (turquoise).

Diffraction patterns show a well-defined β-Co(OH)_2_ structure for the water series, with some samples spontaneously oxidizing under ambient conditions. Bragg peaks can be directly indexed to β-Co(OH)_2_ (ICSD 88940), with the exception of peaks near 11, 23 and 33° (Fig. 1A). The crystal structure contains transition-metal ions linked through di-μ-(hydr)oxo bonds to create 2-dimensional planes. The distance between edge-sharing cations within the 2-dimensional sheets (*d*_MM_) can be calculated by Rietveld refinement (Table S2†), or directly by applying Bragg's law to the (100) or (110) Bragg peak for each sample.^23^ The *d*_MM_ value averages 3.176 ± 0.007 Å across the series, with subtle contraction as Fe-content increases (Fig. 1B). Spacing between layers is directly captured by the (001) peak and is 4.652 ± 0.004 Å across the series (Fig. 1C). Sample storage under ambient conditions results in spontaneous oxidation of W0 through W15 over the span of several days, as evidenced by repeated XRD patterns showing a change from β-Co(OH)_2_ to CoOOH (ICSD 22285, Fig. S4†), and contraction of *d*_MM_ to 2.860 ± 0.003 Å and (001) spacing to 4.428 ± 0.007 Å (Fig. 1B and C). Spontaneous oxidation is well known for Co(OH)_2_ materials with low Fe content.^25–28^ The peak near 11° emerges above W5, which matches the maximum interlayer spacing prior to exfoliation.^29^ Peaks near 23 and 33° grow with increased Fe-content. Similarity to the (001) and (100) peaks, combined with the emergence of a broad feature underneath the (101) feature, suggests formation of CoOOH-like domains with Fe(iii)-incorporation.

Diffraction patterns for the formamide sample series are consistent with Co(OH)_2_ possessing disorder in the *c*-axis. Diffraction patterns across this series show the strong, asymmetric peak near 11°, consistent with a (001) spacing near the limit before exfoliation (Figure 1A),^28,29^ and broad peaks that align with the (001), (100), and (110) of Co(OH)_2_ for α-Co(OH)_2_ (ICSD 86655),^30^ which is a variant of β-Co(OH)_2_ with *c*-axis disorder. The disorder is sufficient to prevent meaningful Rietveld refinements, but the (100) peak shifts with Fe-incorporation to show *d*_MM_ expansion from 3.077 to 3.121 Å between FA0 and FA30 (Fig. 1B). The smooth trends suggest uniform incorporation of Fe(iii) ions into an α-Co(OH)_2_ lattice throughout the series. Energy dispersive X-ray spectroscopy confirms systematic increases in Fe-content across the series (Fig. S3†). Trace chloride content is found in a subset of samples, but is not found to correlate to the sample composition and is expected to rapidly diffuse out of the material when submerged in 1 M KOH solutions.^29^

The Co and Fe K-edge EXAFS spectra reveal non-homogenous distribution of Fe ions in the water series. The Fourier-transformed (FT) spectra contain the two primary peaks expected for metal (oxy)hydroxides (Fig. 1D).^17^ The smaller distance peak captures the average element-specific metal–oxygen bond distances (*R*_MO_), while the other is the di-μ-(hydr)oxo linkages (*R*_MM_). Samples W0 through W15 show dominant *R*_CoM_ and *R*_CoO_ distances of 2.854 ± 0.006 Å and 1.911 ± 0.009 Å, which are shorter than the 3.129 ± 0.012 Å and 2.088 ± 0.031 Å for W20 through W30 (Fig. 1E). Contraction at low Fe-content is consistent with XRD results, confirming that W0 through W15 spontaneously oxidized while awaiting synchrotron measurement. Samples W20 through W30 show compatible *R*_FeM_ and *R*_CoM_ values, but W5 through W15 show significant disagreement between *R*_CoO_ and *R*_FeO_ and between *R*_CoM_ and *R*_FeM_ (Fig. 1E and F). Comparison of *R*_CoM_ to *R*_FeM_, and of each to *d*_MM_, highlights the inconsistency between the Co and Fe coordination environments in the low Fe-content water series (Fig. S5†). The two *R*_MM_ values must be compatible if they coexist within a single structure. Discrepancies therefore indicate incomplete integration of Fe into the Co(OH)_2_ lattice or elemental segregation for the low Fe-content samples in the water series.

The EXAFS of the formamide series indicate that Fe-ions uniformly integrate into Co(OH)_2_. The FT spectra again show characteristic peaks for metal (oxy)hydroxides (Fig. 1D). The locations and intensities for the *R*_FeM_ and *R*_CoM_ peaks are in good agreement with each other across the series (Fig. 1D), confirming uniform distribution of Fe throughout a Co(OH)_2_ lattice. The *R*_FeO_ and *R*_CoO_ features show comparable location for these samples, but *R*_CoO_ is consistently less intense than *R*_FeO_. Both *R*_CoO_ and *R*_FeO_ are therefore comparable on average, but *R*_CoO_ shows measurably more variation. Simulations of the data indicate that *R*_CoM_ values are within 0.01 Å of *d*_MM_ values for this series, while *R*_FeM_ increase from −0.01 Å *vs. d*_MM_ to 0.03 Å (Tables S3–S6, Fig. S1 and S4†). These features suggest that the Co–O bonds distort to accommodate rigid Fe–O bonds and maintain the integrity of the 2-dimensional lattice.

**Fig. 2:**
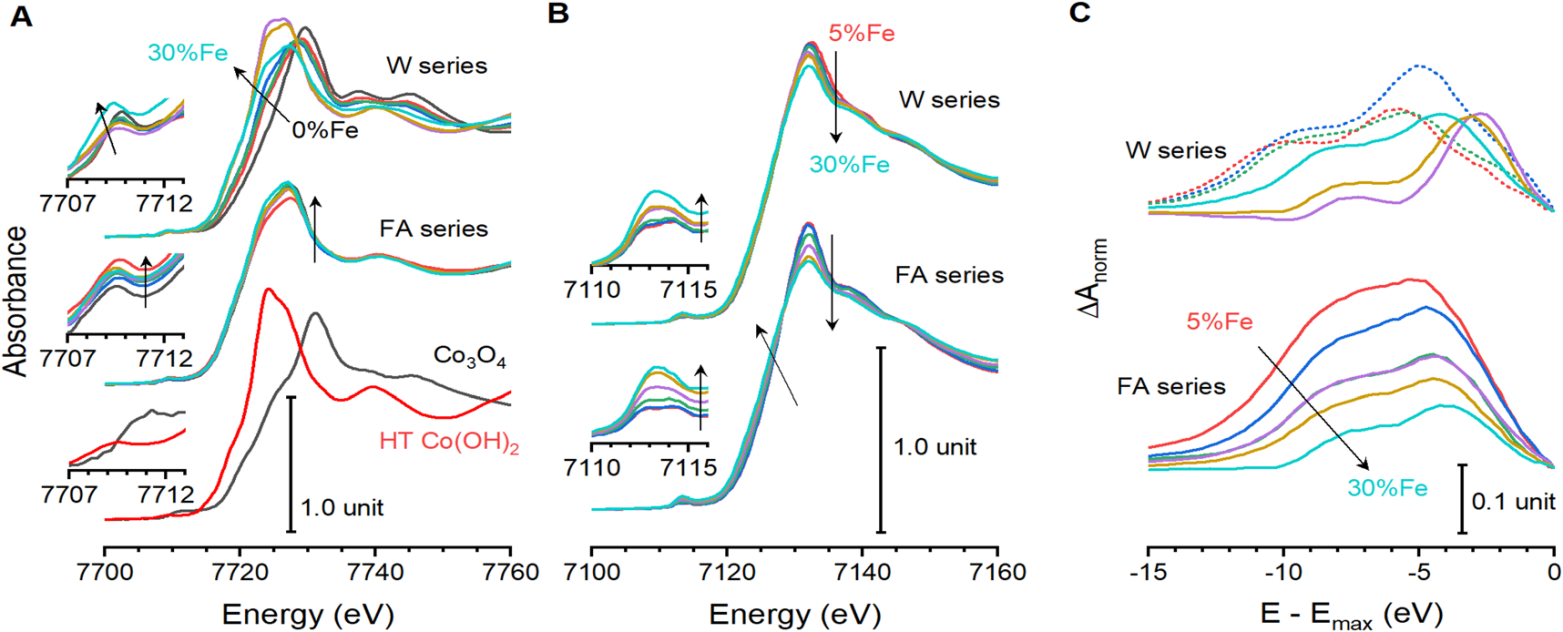
X-ray absorption near-edge spectra for Co_1−*x*_Fe_*x*_(OH)_2_. XANES spectra for the (A) Co K-edge and (B) Fe K-edge of the water (top) and formamide (bottom) sample series. Data is shown for Co_3_O_4_ and hydrothermally synthesized β-Co(OH)_2_ for comparison. (C) Difference plots between the Fe and Co K-edge spectra acquired after normalizing and aligning white line maxima. Arrows depict direction of change with increasing Fe-content. Dotted lines are used to highlight samples W5, W10 and W15, which spontaneously oxidize. Each composition series contains Fe contents of 0% (black), 5% (red), 10% (blue), 15% (green), 20% (purple), 25% (brown), and 30% (turquoise).

Pre-edge features of K-edge spectra are associated with symmetry-forbidden 1s-to-3d excitations, which are visible when distortions relax selection rules.^31^ The formamide series shows composition-dependent changes for Co and Fe: the Co data contains a 7709 eV peak whose intensity increases with Fe-content (Fig. 2A), while Fe shows overlapping peaks at 7112.7 and 7114.2 eV that merge into one at 7113.4 eV that grows with Fe-content (Fig. 2B). The water series shows little change between W0 and W15, with a static peak near 7710 eV for Co (Fig. 2A) and two overlapping peaks for Fe (Fig. 2B). The Co pre-edge feature abruptly shifts to 7709 eV at W20, then shows the same composition dependent changes as the formamide series. This behavior is mirrored in the Fe data, where the peaks merge into a single feature that grows with Fe-content. Intensity growth in pre-edge peak is consistent with increasing bonding environment distortions that disrupt local symmetry.^31^ This is consistent with the composition dependent trends in the EXAFS data (Fig. 1).

XANES spectra are commonly used to assess element-specific oxidation states. The K-edge of the formamide sample series is static near 7718 eV (Fig. 2A; Table S7†), suggesting divalent cobalt ions.^17^ The water series contrasts, showing changes in edge-shape and location. The 7721.0 eV K-edge location measured for W0 is close to values reported for LiCoO_2_ (7721.2 eV) and CoOOH (7721 eV).^32^ The K-edge continually shifts to lower energies between W5 and W15, ultimately stabilizing near 7718 eV for W20 through W30 (Fig. 2A). This analysis confirms that cobalt ions are distinctively different for W0 through W15 than for all other samples.

The Fe K-edges show a decrease in peak intensity in the water series, and changes in edge shape and location in the formamide series (Fig. 2B; Table S7†). A voltage-induced shift in Fe K-edge location was previously reported for electrodeposited Co_0.6_Fe_0.4_(OH)_*y*_ and Co_0.8_Fe_0.2_(OH)_*y*_ using *operando* XANES experiments, which was assigned to partial oxidation of Fe ions.^19^ The composition-induced changes seen in the formamide series are reminiscent of behavior previously seen for Ni_1−*x*_Fe(OH)_2_, however, where a shift in Fe K-edge location was attributed to distortions in bonding environment inducing a change in shape of the spectrum.^23,24^ Local bonding geometry affects electronic structure, which affects the shape and location of K-edge spectra without altering oxidation states.^33–39^ For example, the Zn(ii) K-edge is shifted and distorted by local geometry when ions adsorbed to silica and quartz,^33^ changes in coordination symmetry for Cu(ii) ions distort the rising edge portion of spectra,^37^ and even the fluorine K-edge spectra show variations with changes in local symmetry in the highly-ionic alkali and alkali earth salts.^38^ Simple analysis of edge location cannot differentiate between the two possibilities.

Direct comparison of the Fe and Co XANES spectra that do not spontaneously oxidize reveals systematic changes in relative edge-shapes. The orbitals for Fe and Co ions residing in identical crystallographic environments would experience identical crystal field splittings. They should therefore produce very similar absorption spectra due to their proximity in the periodic table, but with subtle differences due to changes in transition metal electronegativity. The validity of this can be seen using calculated data for Fe and Co: XANES spectra calculated for β-Co(OH)_2_ (ICSD 88940) and one with identical geometry, but with all Co substituted for Fe, show very similar edge profiles (Fig. S6†).^40^ Each spectrum was shifted and normalized such that the white line maxima were aligned with unit absorbance to enable direct comparison of the spectra. This processing effectively defines the energy scale relative to the 4p orbitals of the transition metal, and the intensity relative to the strongest 1s-to-4p transition component. A difference plot derived from these adjusted spectra contains two significant peaks within the rising-edge region: one located 4.1 eV below the white line maximum with *ca*. 10% maximum intensity, and one 9.5 eV below the white line with *ca*. 4% maximum intensity. Being within the edge, these features are attributed to variations in electronic transitions that are primarily 1s-to-4p in nature. We sought to probe the differences in valence structure surrounding the Fe and Co ions by similar direct comparison of the Fe and Co K-edges for each experimental sample (Fig. 2C and S6†). The formamide series and samples W20 through W30 show striking similarities to the calculated spectra, with broad peaks emerging *ca*. 4 and 9 eV below the absorption maxima. These peaks decrease in intensity and a shift towards the absorption edge as Fe-content increases (Fig. 2C). Samples W0 through W15, which spontaneously oxidize, once again present contrasting results with difference peaks *ca*. 3 eV further from the absorption edge. The differences between the oxidized samples and the others shows that changes in transition metal oxidation state have different effects on K-edge structure than compositional changes. The systematic energy shift for difference peaks in the formamide series and W20 through W30 thus appears to be linked to electronic structure modifications. Comparison of XANES behavior with XAFS and XRD leads us to attribute K-edge shifts to composition-dependent changes in bonding rather than changes in oxidation state, with the exceptions of W0 through W15.

**Fig. 3:**
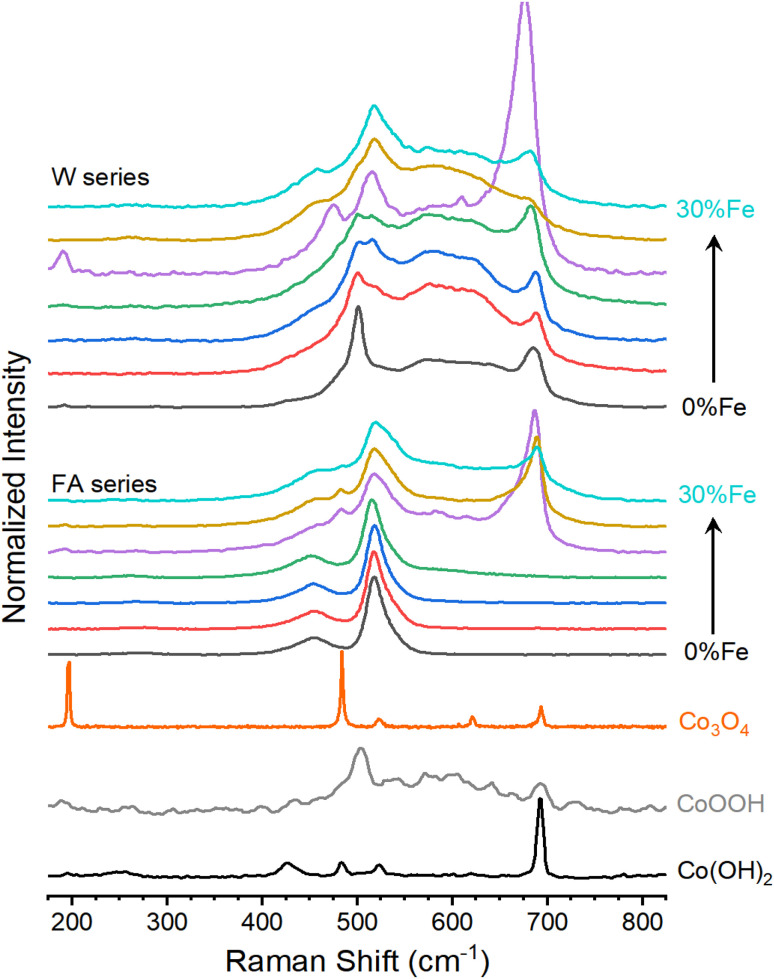
Raman spectra for the Co_1−*x*_Fe_*x*_(OH)_2_ composition series. The water series (top) and formamide series (middle) were normalized to the peak near 500 cm^−1^. Spectra for hydrothermally synthesized Co(OH)_2_, an electrochemically oxidized form of it (CoOOH), and Co_3_O_4_ are provided for comparison. Each composition series contains Fe contents of 0% (black), 5% (red), 10% (blue), 15% (green), 20% (purple), 25% (brown), and 30% (turquoise).

Raman spectra acquired across the formamide series are consistent with a blended metal hydroxide and metal oxyhydroxide structure. Numerous reports on Raman spectra of cobalt (oxy)hydroxides exist, but assignment of spectroscopic features can be inconsistent.^41–47^ A recent report analyzing the transformation of β-Co(OH)_2_ to LiCoO_2_ contains well-defined spectra for β-Co(OH)_2_, CoOOH and Co_3_O_4_ with associated XRD.^48^ The report shows (i) Raman vibrations at 250 (weak), 431 (moderate), and 503 (strong) cm^−1^ for β-Co(OH)_2_, (ii) a broad feature between *ca*. 400 and 640 cm^−1^ and peaks at 505 (moderate), 597 (moderate), and 640 (weak) cm^−1^ for CoOOH, and (iii) peaks at 196 (strong), 493 (strong), 532 (weak), 616, (weak) and 697 (moderate) cm^−1^ for Co_3_O_4_. The formamide series spectra are consistent with β-Co(OH)_2_ between FA0 and FA15, with peaks near 517 cm^−1^ (strong), 453 cm^−1^ (moderate) and 260 cm^−1^ (weak; Fig. 3). The series develops features reminiscent of CoOOH at higher Fe-content: a strong peak emerges near 687 cm^−1^, with a broad feature between it and that at 517 cm^−1^. XRD and XAFS indicate no lattice contraction. These changes are accompanied by loss of intensity of O–H stretching modes (Fig. S7†). The emergence of the *ca*. 517 and 687 cm^−1^ features are therefore attributed to Fe(iii) within the lattice, which induces deprotonation of hydroxide groups due to the cation valence change.^49^ Samples FA20 and FA25 show weak peaks aligned with the major peaks for Co_3_O_4_ at 190 and 482 cm^−1^. No Bragg peaks for Co_3_O_4_ were observed in XRD patterns, suggesting a small degree of laser-induced phase transition to Co_3_O_4_. The Raman spectra are consistent with the conclusion that Fe(iii) ions uniformly integrate into a Co(OH)_2_ matrix.

Raman spectra for the water series show the characteristic behavior of CoOOH, with major peaks near 500 and 685 cm^−1^ and a broad feature between (Fig. 3). A distinct peak appears near 450 cm^−1^ for W20 through W30, which matches one seen in the formamide series that is the major peak expected for Co(OH)_2_. The formamide series and the high concentration water series samples thus exist in similar structures and can be directly compared, while spontaneous changes in the low Fe-content samples in the water series impede direct comparisons.

**Fig. 4:**
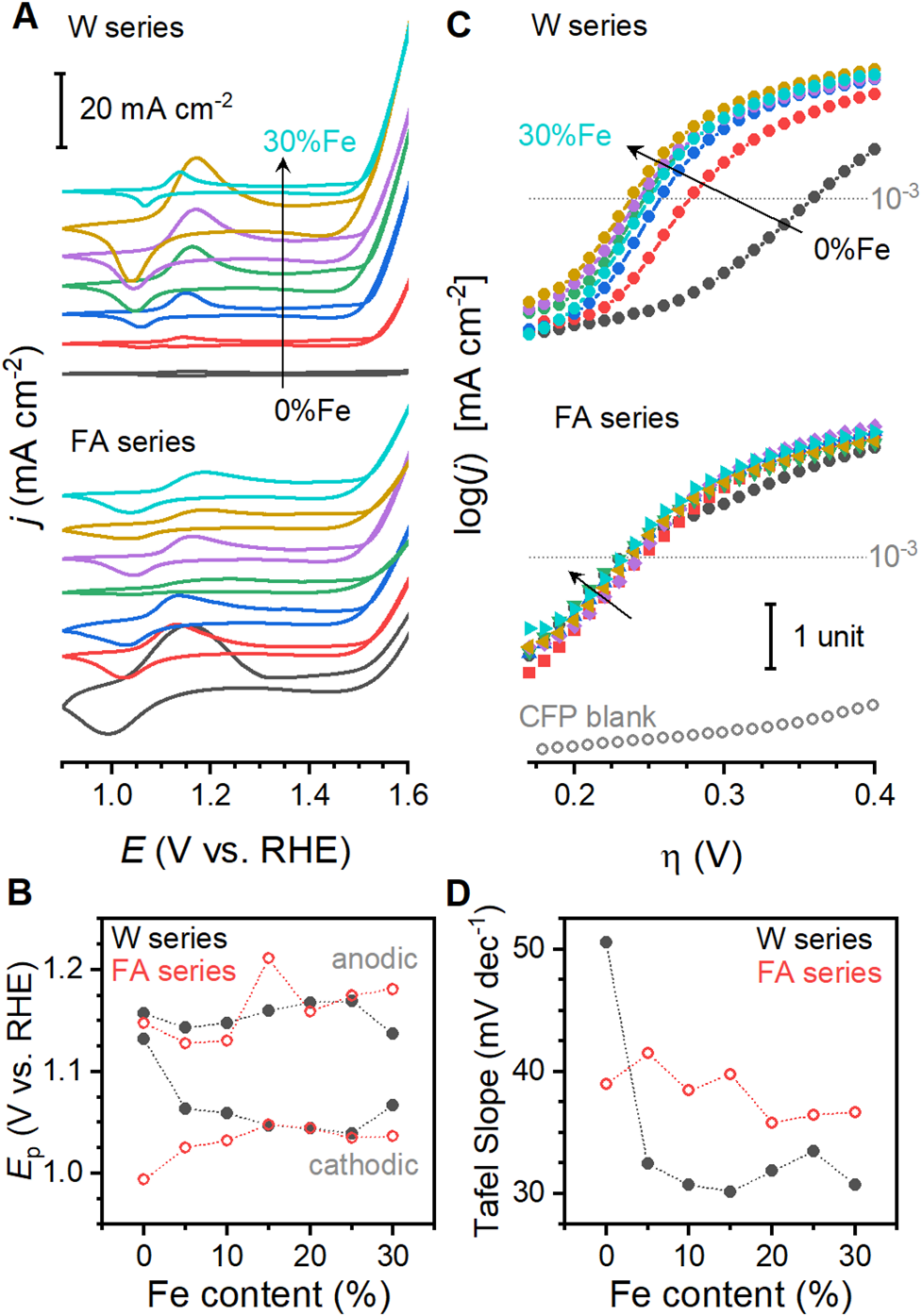
Electrochemical behavior of Co_1−*x*_Fe_*x*_(OH)_2_ sample series. (A) Cyclic voltammograms of water (top) and formamide (bottom) sample series. (B) Composition dependent trends in anodic and cathodic peak locations. (C) Semi-logarithmic steady state current density plots for the water (top) and formamide (bottom) sample series. Data for blank carbon fiber paper substrates are included for comparison. (D) Composition dependence of Tafel slope. Currents are normalized by electrode geometric surface area. Each composition series contains Fe contents of 0% (black), 5% (red), 10% (blue), 15% (green), 20% (purple), 25% (brown), and 30% (turquoise).

Cyclic voltammograms show a prominent reversible redox process in both sample series. With an anodic peak near 1.15 V *vs.* RHE and a cathodic peak near 1.04 V, the process can be assigned as a Co(ii)/Co(iii) transition (Fig. 4A). The anodic and cathodic peaks in the formamide series both trend anodically with Fe-content (Fig. 4B). The anodic peak in the water series behaves similarly, but the cathodic peak initially shifts cathodically, increasing peak separation for the low Fe-content samples that spontaneously oxidize, before shifting anodically. An abrupt decrease in peak spacing for W30 may be associated with partial segregation of iron (oxy)hydroxide, as has been reported for bimetallic metal (oxy)hydroxides.^50^ All samples begin to catalyze OER near 1.4 V. Semi-logarithmic plots of steady state current density against voltage (Fig. 4C) provide a number of parameters that could be utilized for kinetic analysis and benchmarking. We select the Tafel slope, which is extracted from the linear segment of the data (Fig. S8†), as the parameter of interest because it captures the primary change that occurs as a function of catalyst composition and is not affected by normalization procedures (see ESI Discussion S1†). The slope is between 30 and 50 mV dec^−1^ for all samples, consistent with reports on materials with similar compositions.^45,51,52^ The slope gradually shifts to lower values with increased Fe-content in the formamide series, but a much more abrupt change occurs in the water series (Fig. 4D).

## Discussion

**Fig. 5:**
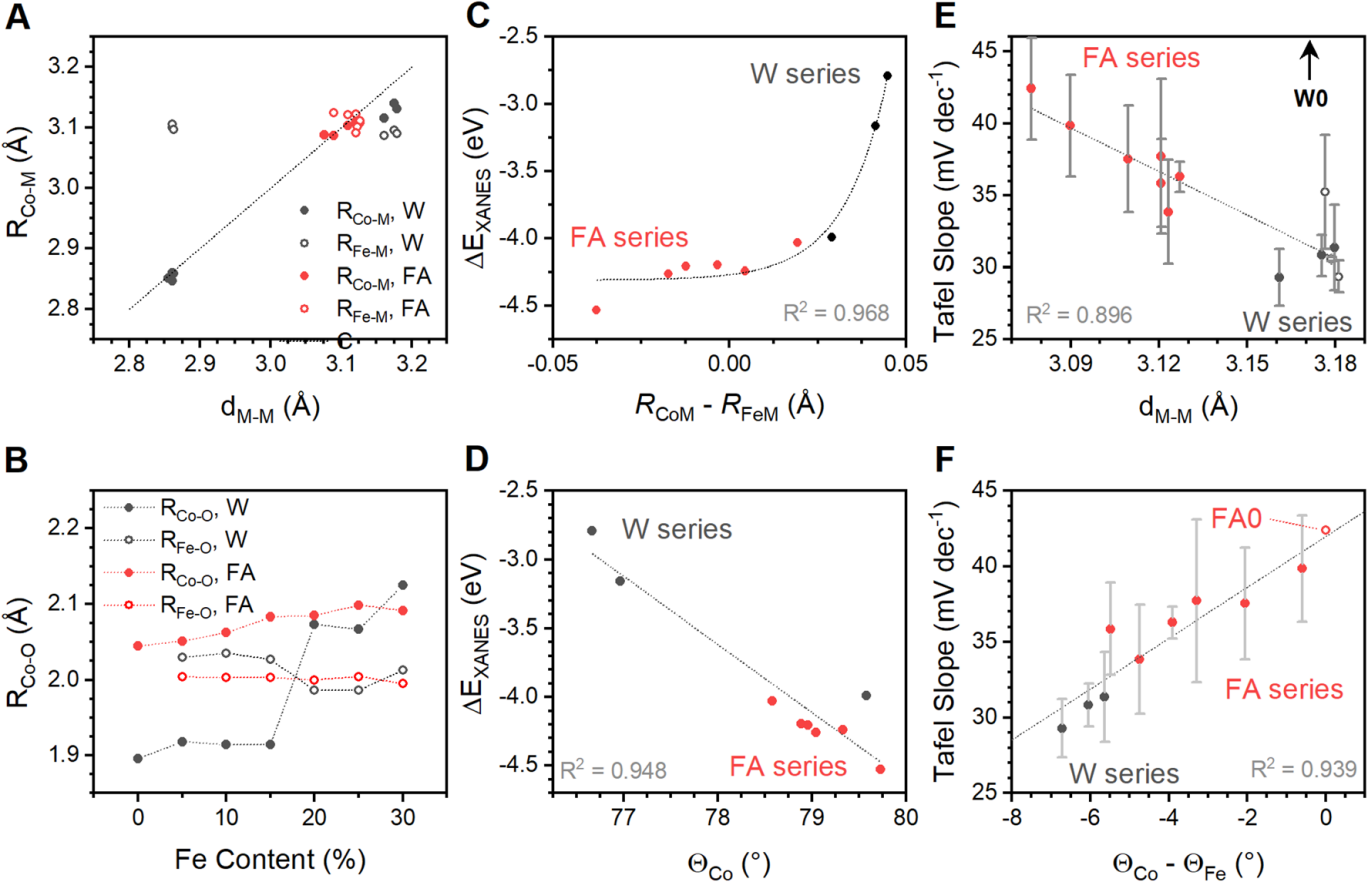
Correlational analysis of structural features in the Fe_*x*_Co_1−*x*_(OH)_2_ sample series. (A) Comparison of M–M distances across di-μ-hydroxo bridges as measured by XRD (*d*_M–M_) and XAS (*R*_M–M_). A line with unity slope and an intercept of zero is drawn to show perfect correlation. (B) Changes in dominant Co–O and Fe–O bond lengths extracted from EXAFS simulations for the two sample series. Relationships between the energy of the primary peak in differential XANES spectra and (C) the Co–M and Fe–M distances across di-μ-hydroxo bridges and (D) the O–Co–O bond angle calculated from EXAFS data. (E) Relationship between measured Tafel slope and the average M–M distance across di-μ-hydroxo bridges prior to spontaneous oxidation of samples W0 through W15 (plotted with hollow datapoints), as measured by XRD. Sample W0 is far removed from others and was not included in calculation of the trend line. (F) Relationship between Tafel slope and the difference between O–M–O bond angles for Co and Fe centers. The FA0 sample was plotted using an angle difference of zero to represent no distortion.

Diverse structural techniques reveal similar structures for all Co_1−*x*_Fe_*x*_(OH)_2_ samples studied, but variations in average coordination environment of Fe and Co. Composition-dependent changes in *R*_CoM_ and *R*_FeM_ confirm incorporation of Fe into the cobalt lattice (Fig. 1), and no signs of phases other than metal (oxy)hydroxides are seen in XAS, XRD or Raman spectroscopy (Fig. 1 and 3). The single *d*_MM_ value derived from XRD presents a model where Co and Fe ions share occupancy of a single crystallographic site, but the mismatch between *d*_MM_, *R*_CoM_ and *R*_FeM_ reveal element-specific distortions within this site (Fig. 5A). The cause of such distortions are seen in metal–oxygen bond lengths: *R*_CoO_ changes with Fe-content while *R*_FeO_ remains static (Fig. 5B). The *R*_CoO_ values are consistently larger than *R*_FeO_ in the formamide series, with continuously expanding *R*_CoO_ values increasing the difference in coordination environments for the two elements. The difference is inverted for spontaneously oxidized samples of the water series then re-established for the high Fe-content samples, as would be expected from ionic radius arguments using Co(ii), Co(iii) and Fe(iii). These trends depict element-specific coordination environment variations due to synthetic procedures and shows composition to be an inadequate structural descriptor.

**Fig. 6:**
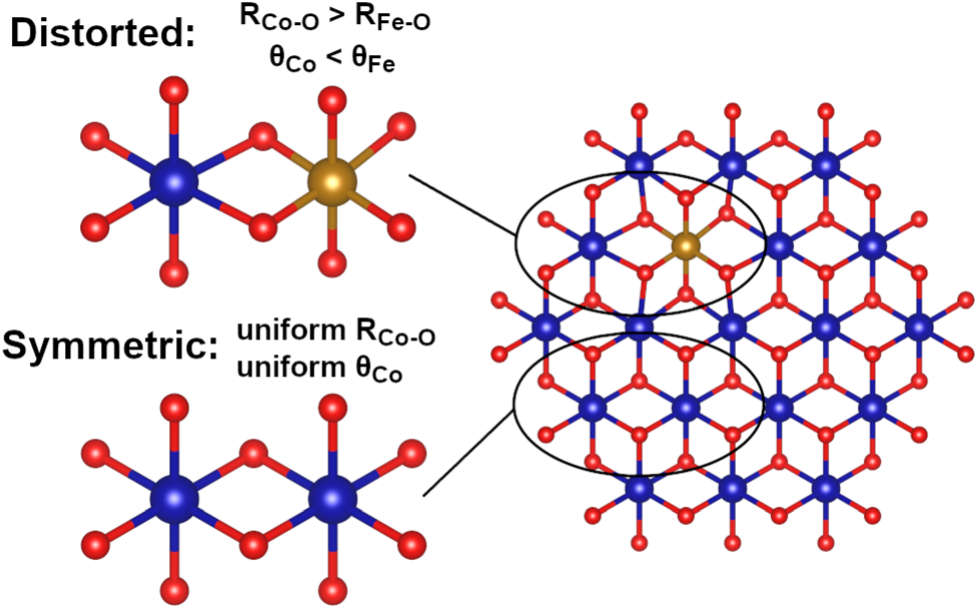
Representation of angular distortion measured around Fe ions embedded within a Co(OH)_2_ lattice. The O–M–O bond angles are distorted in element-specific fashion due to *R*_Fe–O_ being shorter than *R*_Co–O_.

A structural parameter capable of accurately describing samples synthesized by different protocols must capture the relative differences between Fe and Co. The *ca*. 11% compression in lattice parameters upon spontaneous oxidation of Co ions in samples W0 through W15 (Fig. 1) significantly alters strain and bonding considerations for the samples. Further, it changes the effective resting state of the catalyst. Correlational analysis is therefore limited to samples F0 through F30 and W20 through W30, where changes in electronic and bonding structure are due to structural changes rather than spontaneous oxidation of cobalt (all data available in Table S8†). These samples show relationships between local coordination environments and electronic structure parameters from XANES. The location of the primary XANES difference peak (Δ*E*_XANES_) represents a relative difference in electronic structure between Co and Fe (Fig. 2C). This feature is exponentially correlated to the difference *R*_CoM_–*R*_FeM_ (Fig. 5C). EXAFS-derived bond distances can approximate O–M–O angles (*Θ*_M_) across the di-μ-hydroxo bridges linking transition metal ions (ESI Discussion S2†).^21^ A linear correlation between *Θ*_Co_ and Δ*E*_XANES_ is found (Fig. 5D). These correlations confirm that element-specific distortions systematically alter electronic structure. Despite strong structure–property correlations, neither structural descriptor shows a satisfactory relationship with Tafel slopes. The *d*_MM_ values acquired immediately after synthesis, and therefore before spontaneous oxidation of W0 through W15, provide a measure of the average structure. This parameter tracks measured Tafel slopes (Fig. 5E), with lattice expansion accompanying a decrease in Tafel slope. This parameter, however, fails to capture the variations across the water series. Exploration of more complex descriptors found a single parameter, the difference *Θ*_Co_–*Θ*_Fe_ in the reduced catalyst state, that shows good correlation to Tafel slopes across both series (Fig. 5F). Addition of Fe thus alters the electronic structure of the solid (Fig. 5C and D), with the static nature of *R*_Fe–O_ (Fig. 5B) forcing Co ions to increase their distance from nearest neighbours (Fig. 1B) and decreasing *Θ*_Co_ relative to *Θ*_Fe_ (Fig. 5F). This distortion, graphically represented in Fig. 6, is intimately related to changes in the Tafel slope for electrocatalytic OER, which is the primary feature that improves catalyst performance in these samples. Preferential residence of Fe ions along the edge has been suggested.^53^ The differences in structural parameters between the water series and the formamide series are believed to arise from changes in distribution of Fe-ions within the lattice. Experimental strategies to confidently measure the location and distribution of dopant ions in this family of materials need further development.

A correlation between Tafel slope and relative bond angles appears to reveal failure of a common assumption applied to analysis of electron transfer kinetics. Prevailing electron transfer theories define single-electron elementary reactions where an applied voltage offsets the oxidized and reduced PES, and a symmetry coefficient (*β*_a_) captures the proportional change in activation energy for the reaction (defined from anodic perspective here). Microkinetic models have long demonstrated that the Tafel slope of anodic electrocatalytic reactions can be generalized,^54–57^ where the slope is affected by (i) *β*_a_ of the rate-limiting step if it involves electron transfer and by (ii) the number of electrons transferred prior to the rate-limiting step (*n*_pre_):1
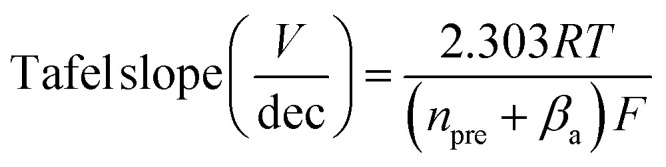


**Fig. 7:**
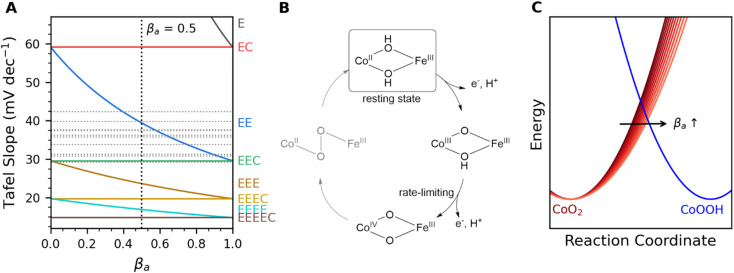
Mechanistic analysis. (A) Comparison of Tafel slopes predicted for combinations of electrochemical (abbreviated E) and chemical (abbreviated C) steps. The terminal character is rate-limiting for each theoretical curve, and experimentally measured values for Fe_*x*_Co_1−*x*_(OH)_2_ are marked as horizontal gray lines. (B) Proposed mechanism, where the second electron transfer from the catalyst rest state is rate-limiting. (C) Depiction of asymmetric distortion of potential energy surfaces that would induce an increase in the symmetry coefficient, *β*_a_.

Factors such as equilibrium constants of chemical reactions or adsorption isotherms alter the log(*i*)-intercept of log(*i*)–*E* plots, but not the slope (ESI Discussion S3†).^54–57^ Variations in catalytic mechanism, identity of rate-limiting step, and *β* can produce a range of possible Tafel slopes (Fig. 7A), but possibilities are commonly truncated by assumption that *β* is 0.5 (ESI Discussion S3†). Experimental evidence for varied values exist, however, including reported values between 0.26 and 0.86 for heterogeneous electrocatalysts,^58,59^ changes with applied voltage,^54,60^ and with temperature.^54,61,62^ The experimentally observed Tafel slope values, and their direct correlation with a structural distortion, are incompatible with the simplifying assumption that *β* is 0.5 (Fig. 5F and 7A).

The observed behavior is proposed to arise from oxidation of Co(OH)_2_ to CoOOH followed by rate-limiting oxidation of CoOOH to CoO_2_ (Fig. 7B), with *β*_a_ of the rate-limiting step being altered by structural distortion. Oxidation of Co(OH)_2_ to CoOOH induces significant lattice contraction (Fig. 1). The subsequent oxidation of CoOOH to CoO_2_ would be expected to induce similar lattice contraction. The tensile strain arising from inclusion of Fe ions into the lattice would inhibit such lattice contractions, effectively broadening the PES for the oxidized states (Fig. 7C). The resultant increase in *β*_a_ can be viewed as a decrease in the reorganization energy as defined by asymmetric Marcus-Hush theory.^63–65^ The Tafel slope reaching 30 mV dec^−1^ at the highly distorted end of the correlation suggests that *β*_a_ changes may accelerate the second electron transfer sufficiently to shift rate-limiting nature to subsequent O–O bond formation. Evidence for a lattice oxygen mechanism (LOM) in Co-based oxides has grown in recent years,^64–68^ and the unusually short distance between neighboring lattice oxygen ions in Na_0.67_CoO_2_ has been shown to accelerate OER.^68^ The contraction of *Θ*_Co_ observed here is conceptually consistent with such reports, suggesting that installation of localized distortions using dopants with rigid M–O bonds may be a viable design strategy to induce LOM in electrocatalysts. The dependence of *β*_a_ on both catalyst composition and catalyst fabrication means that samples with the same nominal composition cannot be directly compared across the literature; the ability to correlate performance to a single structural descriptor demonstrates that meaningful comparisons can nonetheless be made if sufficient information is provided.

The mechanistic proposal presented here was derived using structure–property correlations obtained using structural features measured for the bulk materials rather than the surface. It therefore remains unclear what influence surface-based phenomena have on the observed behavior and overall system. It has been demonstrated, for example, that the elemental composition at the surface of layered double hydroxides may be altered relative to the bulk,^69^ that cyclic dissolution/redeposition processes induce dynamic changes to surface composition and morphology,^12,13,70^ that changes in crystallite size can alter catalyst performance,^71,72^ and that redox-driven changes in catalyst conductivity can alter performance.^73^ Our analysis also does not consider what influence emerging alternative mechanisms for electrochemical reactions may have, such as the recent suggestion that charge accumulation at the electrode surface may induce a change in Tafel slope,^74^ and that the pre-exponential term in Arrhenius' handling of reaction kinetics may be important for electrocatalytic reactions.^75^ The analysis of how such surface-based phenomena influence the bulk-derived structure–property correlations may enable development of robust catalyst design principals for use by researchers active in electrocatalysis.

## Conclusions

We demonstrate element-specific distortions in Co_1−*x*_Fe_*x*_(OH)_2_ that vary with both material composition and fabrication protocol. XRD, XAFS and Raman spectroscopy confirm a similar structure for all samples, with successful Fe-incorporation into Co(OH)_2_ confirmed by composition dependent changes in di-μ-hydroxo bridges distances. EXAFS reveals remarkably stable Fe–O bond distances across all samples, which contrasts with composition-dependent trends in Co–O bonds. Correlations between structural parameters from the different characterization techniques show that rigid Fe–O bonds are accommodated by distorting the local Co coordination environment relative to Fe environment. This alters the electronic structure, as measured by XANES, which ultimately improves electrocatalytic OER. The difference in O–M–O bond angles between the Co and Fe sites is a structural feature found to correlate to the kinetic response. Microkinetic analysis suggest that the structural distortion alters the kinetic response through asymmetric distortion of potential energy surfaces for the Co(iii) to Co(iv) redox transition. Identification of a specific structure–property correlation that relates localized distortions to electrocatalyst performance parameters provides a design principle to assist ongoing efforts to rationally design electrocatalysts.

## Methods

### Materials

Cobalt chloride hexahydrate (CoCl_2_·6H_2_O) (Certified ACS Grade, Fisher Chemical), ferric chloride (FeCl_3_) (Purified Grade, Fisher Chemical), sodium hydroxide and potassium hydroxide (NaOH and KOH) (Reagent Grade, Sigma-Aldrich), and formamide (Reagent Grade, Sigma-Aldrich) were used as received. Milli-Q H_2_O (18.2 M Ω) was used for all synthesis and experiments.

### Synthesis

Two analogous layered double (oxy)hydroxide composition series were fabricated using variations of a pH precipitation protocol. Conventional aqueous pH precipitation was carried out by dissolving appropriate amounts of ferric chloride and cobalt chloride hexahydrate in Milli-Q water to obtain a total metal ion concentration of 0.35 M with the desired stoichiometry to obtain Co_*x*_Fe_1−*x*_(OH)_2_, where *x* was varied from 0 to 0.3. A 1 M aqueous NaOH solution was added dropwise to the solution while stirring under N_2_ environment until a pH of 12 was reached. Stirring was maintained for 10 additional minutes before the solid product was collected by centrifugation. The solid was washed twice by suspending in 10 mL aliquots of water, three times in 10 mL aliquots of acetone, then dried overnight at 75 °C. The fabrication protocol was modified for the second sample series by the addition of formamide to the reaction solution, which has previously been reported to produce single-layered sheets by hydrogen bonding to the basal planes of the metal (oxy)hydroxide and disrupting layer stacking.^76^ An identical protocol was employed for this sample series as the aqueous samples, with the exception that the precursor solutions were prepared in aqueous solutions containing 30% v/v formamide. The two-composition series are referred to here as the water series and the formamide series.

Hydrothermal synthesis was used to prepare a β-Co(OH)_2_ sample for comparison. A 40 ml solution containing 0.6 M aqueous solution of cobalt chloride hexahydrate and 5 M potassium hydroxide was added to a Teflon autoclave. The autoclave was heated at 140 °C for 16 hours. The supernatant liquid was then removed, solid particles were washed with Milli-Q water, and the solid was added into an autoclave reactor again with 40 ml of Milli-Q water. The reactor was heated to 170 °C and held for 16 hours. The final product was collected by vacuum filtration, rinsed with water then ethanol, then dried in an oven at 200 °C for two hours. A nanocrystalline form of Co_3_O_4_ was synthesized using a previously published protocol.^77^

### X-ray absorption spectroscopy

XAS experiments were performed at the Beamline for Materials Measurements (BMM) at the National Synchrotron Lights Source II (Brookhaven National Laboratory, NY, USA) using a Si(111) monochromator. Data were collected in transmission mode for the Co K-edge of all samples, and in both transmission and fluorescence mode, using a 4 element Si drift detector, for Fe K-edge data. The Athena software package was used for data reduction of XAS spectra using standard processes.^78^ The Artemis software package was used to generate structural models by simulation of k^3^ weighted EXAFS results between *k* values of 3 and 15 Å^−1^. Considering similarities in nominal structure, the data was fitted using a group-fitting strategy across the sample series. The Debye–Waller factor (*σ*^2^), the amplitude reduction factor (*S*_o_^2^), and *E*_o_ were fixed for all M–O and M–M shells for the Co and Fe K-edges. The bond distances, R_M–O_ and R_M–M_, and coordination numbers, N_M–O,_ and N_M–M_, were then fitted for each sample in the composition series (Tables S3–S6†). Samples that showed unreasonably low coordination numbers were fitted using two unique M–O and M–M values. The validity of this approach is supported by the coordination numbers of the two M–O shells summing to near an expected value of 6 for the layered double (oxy)hydroxide structure, and observation of structures resembling Co(OH)_2_ and CoOOH in other analytical techniques. The location of Co and Fe K-edges were determined by half-height method (Table S7†).

### Electrochemical analysis

Experiments were conducted in a single compartment polyethylene cell using a Biologic SP-300 electrochemical workstation. A Gaskatel HydroFlex reversible hydrogen electrode (RHE) was used as reference electrode, platinum mesh as the counter electrode, and Toray carbon fiber paper as a substrate for the working electrode. A total of 5 mg of each powdered sample was dispersed in a liquid containing 780 μL of ethanol, 200 μL of ethanol of H_2_O, and 20 μL of 0.5% Nafion suspension by ultrasonication, then a 20 μL aliquot was deposited on a carbon fiber paper electrode. All data is displayed following normalization by geometric surface area of the electrode. We note that this does not enable quantitative analysis of specific activity of the catalysts, but that the primary parameters analyzed in the manuscript are not affected by normalization procedure. A 1 M KOH solution served as the electrolyte. Trace-level contamination of ferric ions in alkaline solutions are well documented to integrate into Fe-free materials such as Co(OH)_2_ and Ni(OH)_2_, substantially altering their electrochemical properties.^69,79^ Such changes have not been reported or systematically studied for materials with non-zero Fe-content, however, such as the Co_1−*x*_Fe_*x*_(OH)_2_ series studied here. Such Fe-containing materials will activate cyclic dissolution/deposition processes for the Fe-ions,^12^ ultimately negating the purification step by transferring Fe ions into solution. Electrolyte solutions were therefore used in their as-prepared state, without purification of trace Fe-contaminants. Cell resistance values on the order of 5 Ohms were recorded at open circuit potential at the onset of each experiment. Cyclic voltammetry experiments were recorded at 10 mV s^−1^ between 0.9 and 1.7 V *vs.* RHE for ten cycles, and the last cycle of each sample was compared. Steady state electrokinetic behavior was analyzed using 60 second chronoamperometry experiments that were recorded in 10 mV between 0.9 V and 1.7 V. Measurements were repeated in both anodic and cathodic directions. Tafel slopes were calculated by fitting the linear portion of semi-logarithmic log(*i*)–*E* plots (Fig. S8†), and the average between the two is used for analysis. Error bars represent the standard deviation between these values.

### X-ray diffraction

Diffraction patterns were measured by using a PANalytical Empyrean diffractometer with Cu K_α_ radiation (*λ* = 1.5405 Å) and 2*θ* angles between 10 and 80° at a step size of 0.0250°. The Bragg–Brentano geometry equipped with a PIXcel bidimensional detector and a Ni foil filter. Rietveld refinements were performed on the water series of samples using GSAS-II software (version 5684). A polynomial baseline was used and parameters refined included scale factor, sample displacement, unit cell parameters, and crystal domain size. A uniaxial domain was used for crystallite size, with the (001) axis defined as the unique axis. Refinements were not possible on the formamide sample series due to the high degree of disorder.

### Raman spectroscopy

Raman measurements were performed using a Renishaw inVia Reflex confocal Raman microscope. All phases of cobalt oxides and (oxy)hydroxide materials are susceptible to laser-induced phase transition to Co_3_O_4_, including CoO, Co(OH)_2_, and CoOOH.^80,81^ Tests were carried out to identify a suitable laser power to avoid phase transitions. A 532 nm (Renishaw DPSSL, 50 mW) laser, filtered to 1% of maximum intensity unless otherwise stated, was used in conjunction with 1800 lines/mm diffraction grating. Raman data were processed and analyzed by Renishaw WiRE 5.3 software package. Processing of spectra includes subtraction of baseline, spectrum normalization, and curve fitting.

### Energy dispersive X-ray spectroscopy

Measurements were performed using an Oxford X-Max attached to a LEO 1530 electron microscope. An incident energy of 20 kV was focused on powder-form samples, which were attached to double sided carbon tape and mounted on aluminum substrates. Elemental content was analyzed using the X-max Aztec software package.

## Author contributions

Conceptualization, formal analysis, and writing: EPA and RDLS; investigation: EPA, MB, JRB, and RDLS; funding acquisition and supervision: RDLS.

## Conflicts of interest

The authors declare no conflict of interest.

## Supplementary Material

SC-015-D4SC01841A-s001

## Data Availability

The data that support the findings of this study are available from the corresponding author upon reasonable request.
