# Supplementary material for: Interplay between element-specific distortions and electrocatalytic oxygen evolution for cobalt–iron hydroxides
Source: Chem Sci. 2024 Aug 27;15(37):15339–51. doi: 10.1039/d4sc01841a (PMC11367221; doi:10.1039/d4sc01841a)
Supplement: SC-015-D4SC01841A-s001 [file SC-015-D4SC01841A-s001.pdf]

Supplementary Information (SI) for Chemical Science.
This journal is © The Royal Society of Chemistry 2024

---SUPPLEMENTARY INFORMATION---

Interplay Between Element-Specific Distortions and Electrocatalytic Oxygen Evolution for Cobalt-Iron Hydroxides

Elif Pınar Alsaç[a], Marlyn Boke[a], Justine R. Bissonnette[a], and Rodney D. L. Smith[a,b,c]*

[a] Department of Chemistry, University of Waterloo, 200 University Avenue W., Waterloo, Ontario, Canada N2L 3G1

[b] Waterloo Institute for Nanotechnology, University of Waterloo, 200 University Avenue W., Waterloo, Ontario, Canada N2L 3G1

[c] Waterloo Artificial Intelligence Institute, University of Waterloo, 200 University Avenue W., Waterloo, Ontario, Canada N2L 3G1

Correspondence to:

rodsmith@uwaterloo.ca

Supplementary Discussion 1: *Catalyst Performance Parameters*

Benchmarks and Behavior Parameters. Comparisons of catalyst behavior between labs is reliant on compatibility of experimental protocols and selection of behavior parameters to report. Electrocatalytic reactions are characterized by the ability to increase reaction rate by increasing electrochemical voltage in the cell. This provides significant tunability in reactions, but also complicates performance reporting. The selection of experimental methodology and the selection of parameters to report have therefore been the subject of much recent interest. Recent publications on technique selection have, for example, highlighted the need to use potentiostatic techniques to accurately measure electron transfer,[1] the desirability of using multiple experimental techniques,[2] and shown that stability in one type of electrochemical cell may not translate to other designs.[3,4] A desire to benchmark catalyst performance in a way that is compatible between different labs has become a topic of debate, with much attention on the protocol used for normalization of currents. Variations are commonly seen across the literature, including normalizing currents by electrode geometric area, electrochemically active surface area, or measured physical surface area. Recent publications show that normalization by mass of Co in Co (oxy)hydroxides,[5] by mass of Ir in IrO_2 particles on a surface,[6] and by surface area of monodisperse RuO_2 particles on a surface[7] all showed variations in benchmark performance parameters as a function of secondary considerations such as particle size. This highlights an important assumption that is implicitly made when comparing catalysts: every normalization approach inherently assumes that the chosen normalization area is correlated to the number of catalytically active sites. The examples above show that even with the best intentions, users can introduce additional uncertainty into results. Within this work, we analyze the Tafel slope rather than single value parameters such as the popular voltage-at-current type parameters. As is discussed below, the slope of semi-logarithmic log(i) versus E plots is not affected by normalization. This approach provides a reliable numeric value that can be easily compared to structural features measured in the samples.

Elementary electrochemical reactions. Electron transfer theories ranging from the classic Butler-Volmer equation through to the more modern asymmetric Marcus-Hush model treat electrochemical reactions as single electron elementary steps that exhibit an exponential relationship between current (i) and applied voltage (E). The most widely used mathematical description of this relationship arises from the current-overpotential equation, derived using the Butler-Volmer approach:

$$\ln(i) = \ln(i_0) + \beta_a \frac{RT}{nF}\eta \quad \text{(S1)}$$

Where the exchange current (i_0) is measure of kinetic facility of the electron transfer, η is the overpotential defined relative to the equilibrium of the system (η), β_a is the symmetry coefficient that is an empirical fraction that conceptually captures changes in relative symmetry of the potential energy surfaces of the oxidized and reduced species (defined here from anodic perspective, with the understanding that the anodic and cathodic terms sum to unity; $\beta_a + \beta_c = 1$), and *F*, *R* and *T* have their common meaning. This model has been remarkably successful and resilient across decades of research because it provides a direct and simple model to analyze the underlying chemistry: i_0 is related to the standard rate constant and therefore describes the inherent kinetic facility of the reaction, while β provides a measure of relative changes in the shape of potential energy surfaces between the reactant and product. The development of Marcus-Hush and Asymmetric Marcus-Hush models have shown that β, while being an empirical parameter in the Butler-Volmer model, does have physical meaning that is related to the reorganization energy associated with the electron transfer.[8–11]

Multi-step electrochemical reactions. The kinetic parameters present in equation (S1) are not applicable to any situation beyond an elementary 1-electron transfer reaction. Semi-logarithmic log(*i*)-*E* plots continue to show linearity for multi-step electrocatalytic reactions with a single well-defined rate limiting step, but the mathematical descriptions are more complex (see Supplementary Discussion S3). Consider the situation where an electrocatalytic reaction proceeds via an initial electron transfer that is followed by a second, rate-limiting, electron transfer. It has been shown that the equation describing current for an arbitrary electrocatalytic oxidation is:[12–15]

$$i = \frac{nF\, a_{cat}\, k_2^0\, e^{(n_{pre} + \beta_{a,2}) f\eta}}{K_1} \quad \text{(S2)}$$

Where a_{cat} indicates the activity of the catalyst at the electrode surface, *f* is the ratio F/RT, and thermodynamic (K_i) and kinetic (k_i, $\beta_{a,i}$) are given for respective steps. Kinetic parameters in such equations are only present for the rate limiting step (k_2^0 and $\beta_{a,2}$), but thermodynamic parameters are present for the elementary steps preceding the rate-limiting step (K_1). Microkinetic models consistently show this same perspective:[8,15] terms containing exponential

overpotential dependence combine to establish linearity in semi-logarithmic Tafel plots, while all non-exponential terms only affect the y-intercept. This is seen in the logarithmic form of equation (S2):

$$\ln(i)=\ln\left(\frac{nF\,a_{cat}k_2^0}{K_1}\right)+\left(n|pre+\beta_{a,2}\right)f\eta \tag{S3}$$

Overall, this leads to the Tafel slope being a function of (i) reaction mechanism, (ii) the identity of a rate limiting step within the mechanism, (iii) β of the rate limiting step if it is an electron transfer reaction, and (iv) adsorption isotherms. The log(*i*)-intercept is no longer a direct measure of kinetic facility, but is convoluted with thermodynamic parameters for all reaction steps preceding the rate-limiting step.

Selection of performance parameter. It is important to consider the chemical information contained within any given performance parameter, and limitations inherent in the parameter, when selecting which to use. The observed linearity in log(*i*)-*E* plots for both elementary electron transfers and multi-step reactions means that the entirety of the kinetically limited behavior is described by two experimentally measurable features: the Tafel slope and the y-intercept. Despite the behavioral similarities linking equations (S1) and (S2), the slope and intercept of the semi-logarithmic plots for elementary and multi-step electron transfer reactions have different physical meanings. Only the multi-step reaction mechanism situation will be considered in detail here.

The log(*i*)-intercept in Tafel plots for multi-step mechanisms contains a term describing inherent catalytic performance (ie, k^0), but it is not a direct measure of the exchange current or kinetic facility of the rate-limiting step. This intercept is influenced by thermodynamic descriptors of steps preceding the rate-limiting step within a given mechanism. As seen in equation (S2), a change in K_1 will alter the measured log(*i*) intercept. The dependence on such additional thermodynamic terms means that the measured log(*i*)-intercept is also dependent on the active catalytic mechanism. Any changes in the mechanism, or the rate limiting step within a mechanism, will change the number and identity of thermodynamic terms used to describe the intercept. The intercept is also reliant upon the concentration of catalyst and reactants present at the electrode surface. For example, results have shown that the absolute value of the catalytic current can vary as a function of parameters such as particle size, catalyst mass loading, and surface concentration of specific elements.[5–7] Despite the changes in absolute

current values, these examples each show Tafel slopes to be unaffected by these features. Accurate representation of catalyst performance using the y-intercept requires rigorous accounting of local concentrations in some manner. Electrocatalytic current can be normalized in a number of ways to account for changes in concentration. Examples include normalizing by (i) geometric surface area, (ii) mass of catalyst loaded, (iii) electrochemically active surface area, (iv) charge passed during pre-catalytic redox processes, or (v) total molar content of the element of interest on the surface. Each of these approaches has its own specific strengths and weaknesses and, as recently noted by Schmidt et al.,[16] there is currently no satisfactory way to accurately describe the relevant area of an electrocatalyst on an electrode. All approaches are linked in their need to make an assumption, such as the quantified value being proportional to the concentration of catalytically active sites, which introduces risk of introducing error with an unknown magnitude into the data.

The Tafel slope for multi-step reactions contains the kinetic parameter β, but is also altered by the identity of the mechanism and rate limiting step within that mechanism. The Tafel slope has a particular strength in that it looks at a relative change across a dataset, which means that normalization procedures are irrelevant and no errors are introduced by a user's selection of normalization procedure. The catalyst response to overpotential is strongly affected by β, as seen in Figure S1. This figure shows that a theoretical kinetic profile with a log(*i*) intercept of 10^{-9} A and Tafel slope of 30 mV dec^{-1} begins to outperforms one with an intercept of 10^{-7} A and a Tafel slope of 60 mV dec^{-1} after 120 mV overpotential. This demonstrates that the Tafel slope is an important kinetic descriptor that controls catalyst performance. It also demonstrates that the log(*i*)-intercept value can be misleading even when accurately normalized.

Alternative catalyst performance apramefers, such as current density at specified voltages or voltages required for specified current densities, are derived from the Tafel slope and log(i) intercept values and therefore present a convolution of information and limitations from both. Such descriptors can be effectively used to custom-design catalysts for a given purpose (e.g., OER catalysis at 10 mA cm^{-2} to support the photon flux expected for an artificial leaf device), but are misleading for general catalyst design. This is readily seen in Figure S1: the "best" catalyst identified by drawing a horizontal or vertical line depends on where one draws the line.

Challenges in controlling features such as consistent mass loading, portion of powder that is electrochemically accessible, and accurately defining surface area introduce uncertainty into parameters that use current density directly. Variations in catalyst loadings, composition-

dependent changes in catalyst morphology, composition-dependent changes in specific capacitance, and the need to have a conductive supporting electrode all introduce errors into surface area measurements. Equations such as (S2) contain surface area (A) and concentration of the electroactive species (C_T) as coefficients. This introduces uncertainty into the y-intercept value, and therefore into any i-at-η or η-for-i type parameters. This uncertainty disrupts correlational analysis between structural descriptors and arbitrarily selected current or overpotential values. This is particularly problematic for powder-form samples as analyzed here.

With such consideration in mind, we select the Tafel slope as the primary parameter for this analysis because it is not affected by the catalyst loading.

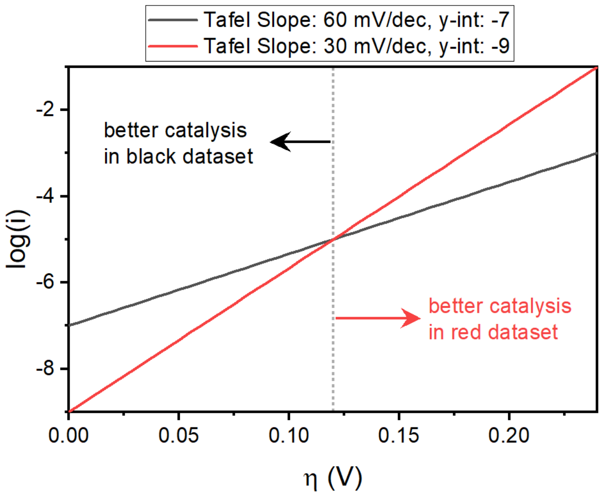

Figure S1. Simulated semi-logarithmic responses demonstrating the effect of a change in Tafel slope on catalyst response.

Supplementary Discussion S2: *Calculation of O-M-O Bond Angles From EXAFS Models*

Data from both XRD and EXAFS indicate that all samples adopt a structure related to the layered double hydroxides (Figure 1). This structure is characterized by MO_6 polyhedra with uniform M-O bond distances. These polyhedra are connected to their neighbors through edge-sharing linkages to form a 2-dimensional framework. The O-M-O bond angle (θ) within this edge sharing linkage is *ca*. 82° in highly crystalline β-$Co(OH)_2$ (ICSD 88940). Within this structure, a line drawn between one transition metal ion (M_1) and a neighbor (M_2) bisects this

bond angle to form a triangle with edges defined as the bond distances $R_{M1\text{-}M2}$, $R_{M1\text{-}O}$ and $R_{M2\text{-}O}$. Application of the law of cosines therefore provides the ability to calculate the bond angle:

$$\cos\left(\frac{\theta}{2}\right)=\frac{R_{M1-M2}^{2}+R_{M1-O}^{2}-R_{M2-O}^{2}}{2R_{M1-M2}R_{M1-O}} \quad \text{(S4)}$$

The values $R_{Co\text{-}M}$, $R_{Co\text{-}O}$, $R_{Fe\text{-}M}$, and $R_{fe\text{-}O}$ are directly measured in EXAFS measurements, and the equation is applicable as long as the XRD model is valid. The fact that Co- and Fe-centered distances are not aligned with one another (Figure 1) reveals the presence of lattice distortions induced by the Fe-dopant ions. The EXAFS results do not formally resolve $R_{Co\text{-}Co}$, $R_{Co\text{-}Fe}$, and $R_{Fe\text{-}Fe}$ distances, so bulk-average values must be used to calculate angles around Co ions (θ_{Co}; M_1= Co, M_2=Fe) and Fe ions (θ_{Fe}; M_1=Fe, M_2=Co). Due to the bulk-average perspective. The angles therefore represent the weighted average of each ion in all possible coordination sites. For example, a Co ion may have 6 Co neighbors, or 5 Co neighbors and one Fe neighbor. The calculated angles are therefore not taken as quantitatively accurate representations of the local distortion. The differences between the Fe-centered and Co-centered angles do, however, provide a measure of the relative magnitude of distortions in the lattice. Due to the weighted averaging, these numbers are expected to systematically underestimate the magnitude of distortion.

Supplementary Discussion 3: *Microkinetic Models and The Nature of Tafel Slope*

Mathematical description of electrokinetic behavior for multi-step reactions was initially explored by Bockris,[13] and revisited over the ensuing decades. These analyses have been described at length in numerous publications, including in Bockris' original work and his subsequent applied research,[14] in a comprehensive report by Lefebvre, in an IUPAC technical report by Trasatti and co-workers, and a more recent demonstration.[8,12,14,15] The detailed mathematical approach is described in detail in the literature,[15] and will not be repeated here. The mathematical strategy, however, is to apply the steady state approximation and define the reaction rate as that for the rate-limiting step. A quasi-equilibrium assumption is made, which stipulates that all steps preceding the rate-limiting step are at a functional equilibrium. This enables the concentration of individual reaction intermediates to be isolated and algebraically represented using equilibrium descriptors. These terms are then substituted into the kinetic equation for the rate-determining step. These past analyses indicate that the Tafel slope is a function of (i) reaction mechanism, (ii) the identity of a rate limiting step within the mechanism,

(iii) the relative symmetry between the potential energy surfaces of the reactant and product of the rate limiting step if that step is an electron transfer reaction, and (iv) possible adsorption isotherms. Despite the seeming complexity, the outcome has been shown to be completely generalized.[15] Specifically, the Tafel slope (in V dec^{-1}) of an anodic reaction given as equation (1) in the main text, and for a cathodic reaction as:

$$TafelSlope = \frac{2.303\,RT}{\left(n_{pre} + \beta_c\right)F} \tag{S5}$$

Where R is the gas constant, T is temperature, F is the Faraday constant, n_{pre} is the number of electrons transferred prior to the rate-limiting step, and β_c is the symmetry coefficient defined from the cathodic reaction perspective. The common assumption that β is 0.5 leads to a series of discrete Tafel slope values that can be linked to combinations of general mechanisms and rate-limiting steps (Table S1; Figure 6). We note that these generalized models only list chemical elementary steps as terminal values. Steps of this nature affect the y-intercept of kinetic plots, but they do not affect the Tafel slope.[8,12,14,15]

Table S1. Tafel slopes predicted for given rate-limiting steps embedded into general catalytic mechanisms.

**Mechanism**[a]	**Tafel Slope ($mV\ dec^{-1}$)**[b]
**E**	118.3
E**C**	59.1
E**E**	39.4
EE**C**	29.6
EE**E**	23.7
EEE**C**	19.7
EEE**E**	16.9
EEEE**C**	14.8

[a] E represents an elementary step involving electron-transfer and C is one not involving electron transfer; the bolded final step is rate limiting

[b] calculated assuming $\beta = 0.5$ and $T = 298$ K

To establish a mechanistic interpretation of the $Fe_xCo_{1-x}(OH)_2$ dataset described in this work, we first consider the primary experimental outcomes:

(i) Steady-state log(i)-E plots show a single linear region for each sample. This region extends from catalytic onset up to current densities where curvature due to mass-transfer limitations are expected (Figure 4C). Analysis is restricted to this linear region, which is a requirement for application of prevailing electron transfer theories.

(ii) The sample series shows measured Tafel slopes between 42 and 30 mV dec^{-1}. With an assumption that β is 0.5, this covers the full range of values between an EE and an EEC mechanism (Table S1).

(iii) The measured Tafel slope values correlate to the difference in measured bond angles for Fe and Co centers within the material.

Formulation of a mechanistic interpretation of the results was pursued by considering phenomena that can alter the Tafel slope and ruling out those that cannot describe a gradient in behavior (as opposed to discrete values as shown in Table S1). Based on prevailing electron transfer theories, options identified include:

(i) *A change in identity of the rate-determining step*. This would result in a step-change in Tafel slope from one discrete value to another. The mathematics of this is clearly described in the literature,[15] and evidence of such a phenomenon has been reported for catalysts such as IrO_2 and PbO_2.[17,18] We find experimental evidence of such phenomena only supports transitioning from a low numeric slope to a higher value.

(ii) *A change in surface coverage of intermediates*. Decades of microkinetic models indicate that this phenomenon will be characterized by a region with a low numeric Tafel slope to a secondary region with a higher numeric Tafel slope. A region of non-linearity would necessarily link the two. It is not conceptually possible for a transition from a high slope to a low slope to occur, as this would signify a move of the thermodynamically stable state of the catalyst (it's "resting state") backwards in the catalytic cycle (i.e., to the last step of the catalyst cycle).

(iii) *A change in catalytic mechanism.* As discussed above, Tafel slopes are linked to reaction mechanisms. A change in reaction mechanism would result in step-change in Tafel slope. The exponential relationship between overpotential and current would lead to a single mechanism dictating observed behavior. A short period of curvature

in log(i)-E plots may arise if a mechanism with a smaller numeric Tafel slope overcomes another, but such a situation is not compatible with the observed gradient in Tafel slopes.

(iv) *A change in oxidation state of the catalyst*. Redox reactions may participate directly as a step within the catalytic reaction cycle, or as a pre-equilibrium step that is inherently required for catalysis. The former is factored directly into the mathematical descriptions indicated in (I). Microkinetic analyses pre-equilibrium steps have been considered in the literature, where the outcome was shown to have the same outcome as situation (i).[19]

(v) *Influence of Adsorption isotherms*. It is possible that formation of a reaction intermediate positively or negatively affects the thermodynamics of subsequent accumulation of the intermediate. This situation is mathematically represented by adsorption isotherms. The influence of Langmuir and Temkin isotherms have been previously considered.[14] The mathematical models indicate that a Tafel slope would to transition between two linear regions, going from a smaller to a larger numeric value as the surface coverage saturates. This behavior is very similar to that seen in situation (i), but with an additional reaction step that effectively changes the generalized mechanism.

(vi) *Two competing pathways, with dominance shifting from one pathway to another.* The two pathways would each be characterized by specific intercept and slope in log(i)-E plots, such the two traces in Figure S1. Due to the exponential relationship between current and voltage, these two pathways would only be simultaneously observed in a short voltage window surrounding the voltage at which they have equivalent current. The behavior would therefore also yield that seen in situation (I): two distinct linear sections in log(i)-E plots that are connected by a region of non-linearity.

(vii) *A change in symmetry coefficient for a rate-limiting electron transfer*. The symmetry coefficient dictates what proportion of applied voltage is used to alter the activation energy of the reaction. This is the only term in prevailing electron transfer theories that can effectively describe a relationship between Tafel slopes and structural features.

Of these phenomena that can affect Tafel slopes, the only one capable of describing the experimental data is option (vii) – a structural modification that causes a gradual change in symmetry coefficient for a rate-limiting electron transfer.

Table S2. Summary of Rietveld refinement results for samples synthesized in the water series.

	a **(Å)**	***c*** **(Å)**	**equatorial size (Å)**	**axial size (Å)**
**W0**	3.181 ± 0.001	4.651 ± 0.001	72 ± 3	56 ± 2
**W5**	3.179 ± 0.001	4.651 ± 0.001	146 ± 15	24 ± 1
**W10**	3.177 ± 0.001	4.648 ± 0.002	70 ± 8	19 ± 1
**W15**	3.177 ± 0.002	4.651 ± 0.003	44 ± 5	18 ± 1
**W20**	3.175 ± 0.002	4.649 ± 0.003	43 ± 5	17 ± 1
**W25**	3.177 ± 0.003	4.658 ± 0.004	21 ± 5	17 ± 5
**W30**	3.177 ± 0.005	4.678 ± 0.009	11 ± 1	19 ± 2

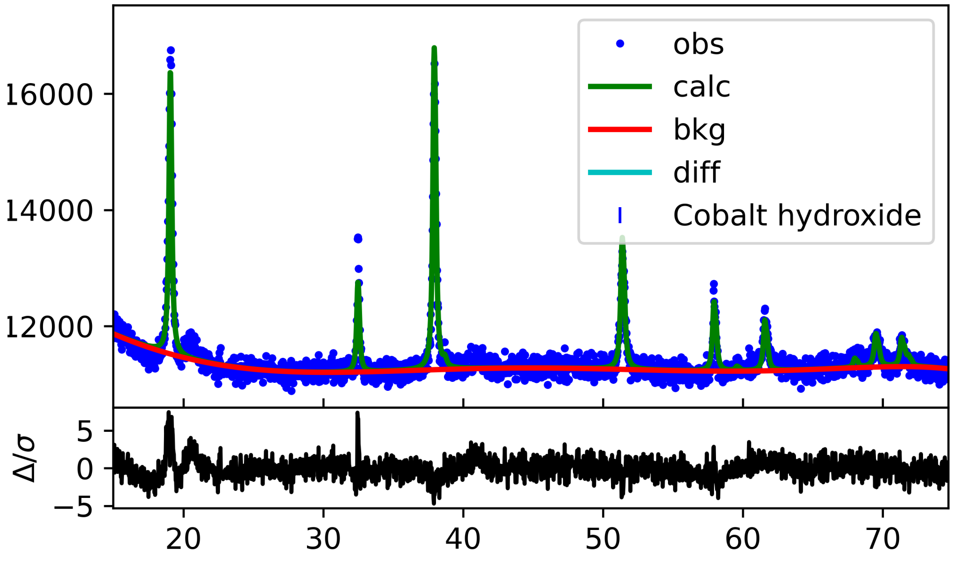

Figure S2. Sample Rietveld refinement, as performed on W0 sample.

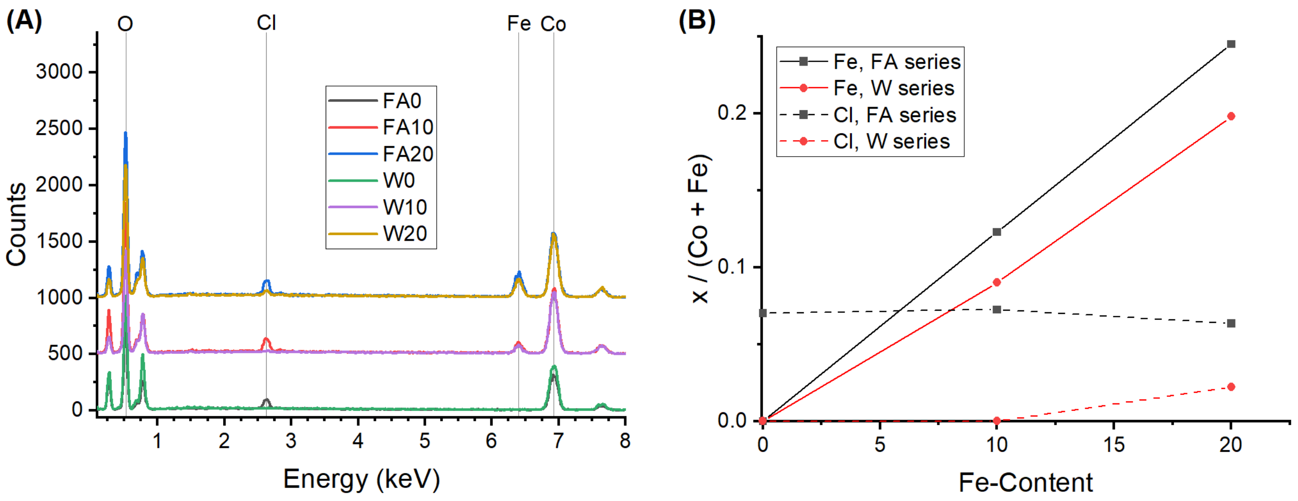

Figure S3. Elemental analysis using energy dispersive x-ray spectroscopy. **(A)** Spectra were measured on W0, W10, W20, FA0, FA10, and FA20. **(B)** The atomic content of Fe and Cl relative to the total metal content of the films.

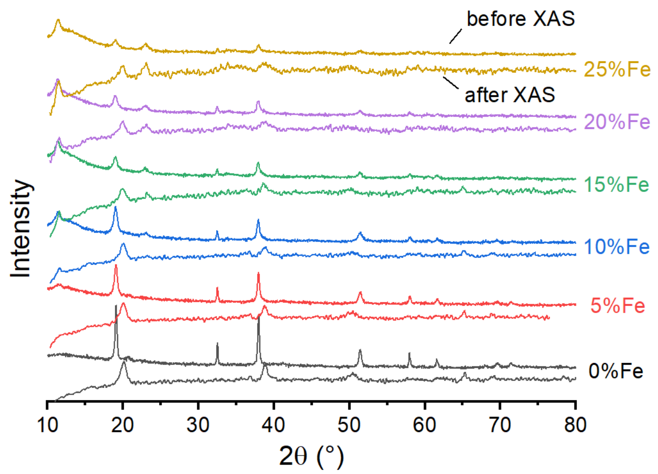

Figure S4. X-ray diffraction patterns acquired on water series $Co_{1-x}Fe_x(OH)_2$ samples after synthesis, then repeated at the start of synchrotron X-ray absorption spectroscopy (XAS) experiments.

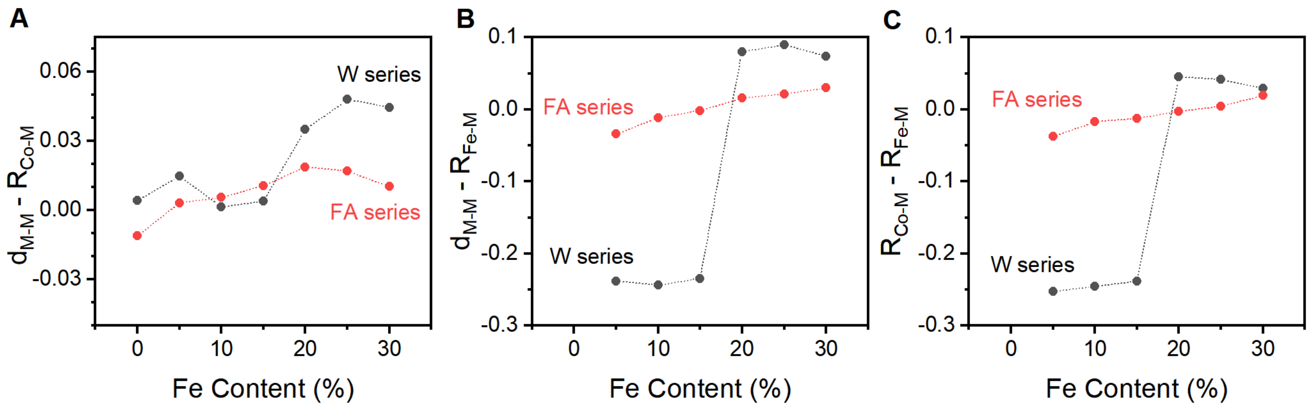

Figure S5. Comparison the M-M distance across μ-di-hydroxo bridges in the two $Co_{1-x}Fe_x(OH)_2$ sample series as measured by X-ray diffraction and X-ray absorption spectroscopy. **(A)** Difference between XRD-derived d_{MM}, which captures an overall average, and EXAFS-derived R_{CoM}, which captures only the host Co ions. The mismatch of R_{FeM} with the host lattice is seen by comparison against **(B)** the average d_{MM} value and **(C)** the R_{CoO} value.

Table S3. Structural parameters acquired by simulation of extended X-ray absorption fine-structure spectra on the Co K-edge of the formamide series.

Fe (%)	N_{M-O}	R_{M-O}	N_{M-M}	R_{M-M}	*R-factor*
**0**	5.5 (0.3)	2.045 (0.007)	6.0 (0.5)	3.088 (0.006)	0.030
**5**	4.5 (0.4)	2.051 (0.009)	5.1 (0.6)	3.087 (0.007)	0.038
**10**	5.5 (0.3)	2.062 (0.006)	5.6 (0.5)	3.104 (0.006)	0.030
**15**	5.0 (0.3)<br>1.4 (0.6)	2.083 (0.016)<br>1.932	4.6 (0.6)<br>1.1 (0.5)	3.110 (0.007)<br>2.855*	0.019
**20**	4.8 (0.2)<br>1.5 (0.6)	2.085 (0.015)<br>1.932*	4.0 (0.6)<br>1.2 (0.5)	3.108 (0.008)<br>2.855*	0.019
**25**	4.9 (0.3)<br>1.8 (0.7)	2.098 (0.019)<br>1.932*	3.7 (0.7)<br>0.8 (0.6)	3.106 (0.011)<br>2.855*	0.033
**30**	5.7 (1.1)<br>1.1 (1.0)	2.091 (0.023)<br>1.932*	5.2 (1.0)<br>1.2 (0.9)	3.110 (0.011)<br>2.855*	0.044

E_0=12.0 eV, $S_O^2=0.78$, $\sigma^2_{M-O}=0.01$, $\sigma^2_{M-M}=0.007$, * fixed parameter

Table S4. Structural parameters acquired by simulation of extended X-ray absorption fine-structure spectra on the Fe K-edge of the formamide series.

Fe (%)	N_{M-O}	R_{M-O}	N_{M-M}	R_{M-M}	E_0	*R-factor*
**5**	6.3 (0.2)	2.004 (0.006)	6.4 (0.5)	3.124 (0.006)	15.6	0.027
**10**	6.2 (0.2)	2.003 (0.006)	6.4 (0.5)	3.121 (0.006)	15.6	0.012
**15**	5.7 (0.3)	2.003 (0.008)	5.3 (0.6)	3.122 (0.009)	15.7	0.018
**20**	5.2 (0.2)	2.000 (0.009)	3.6 (0.5)	3.112 (0.012)	15.7	0.023
**25**	4.4 (0.3)	2.004 (0.014)	3.1 (0.6)	3.102 (0.016)	16.3	0.049
**30**	4.3 (0.3)	1.995 (0.013)	2.8 (0.6)	3.091 (0.0175)	15.7	0.061

S_O^2=0.78, σ_{M-O}^2=0.004, σ_{M-M}^2=0.006

Table S5. Structural parameters acquired by simulation of extended X-ray absorption fine-structure spectra on the Co K-edge of the water series.

Fe (%)	N_{M-O}	R_{M-O}	N_{M-M}	R_{M-M}	R-factor
**0**	6.1 (0.2)	1.896 (0.003)	6.0 (0.3)	2.851 (0.003)	0.009
**5**	4.5 (0.3)	1.918 (0.006)	4.1 (0.4)	2.847 (0.005)	0.020
**10**	4.1 (0.2)<br>2.2 (0.2)	1.914 (0.005)<br>2.128 (0.011)	3.0 (0.2)<br>1.5 (0.3)	2.860 (0.004)<br>3.133 (0.010)	0.004
**15**	4.4 (0.2)<br>1.5 (0.2)	1.914 (0.004)<br>2.200 (0.0150	3.6 (0.2)<br>1.6 (0.3)	2.859 (0.003)<br>3.101 (0.009)	0.003
**20**	5.3 (0.5)	2.073 (0.010)	3.8 (0.8)	3.140 (0.012)	0.086
**25**	5.0 (0.5)	2.067 (0.010)	3.7 (0.7)	3.131 (0.013)	0.090
**30**	3.0 (0.3)<br>2.8 (0.3)	2.125 (0.014)<br>1.945 (0.012)	2.5 (0.4)<br>1.2 (0.3)	3.116 (0.009)<br>2.896 (0.009)	0.014

E_0=11.5 eV, S_O^2=0.78, σ_{M-O}^2=0.004, σ_{M-M}^2=0.004

Table S6. Structural parameters acquired by simulation of extended X-ray absorption fine-structure spectra on the Fe K-edge of the water series.

Fe (%)	$N_{M\text{-}O}$	$R_{M\text{-}O}$	$N_{M\text{-}M}$	$R_{M\text{-}M}$	R-factor
**5**	4.1 (0.7)<br>2.0 (0.8)	1.962 (0.011)<br>2.029 (0.009)	3.2 (0.1)<br>3.7 (0.1)	2.884 (0.011)<br>3.0997 (0.009)	0.013
**10**	4.3 (1.1)<br>1.4 (1.2)	1.972 (0.014)<br>2.035 (0.011)	2.8 (0.7)<br>4.6 (0.9)	2.894 (0.142)<br>3.106 (0.011)	0.032
**15**	3.3 (0.8)<br>2.4 (0.9)	1.958 (0.014)<br>2.027 (0.008)	2.3 (0. 6)<br>5.4 (0.7)	2.880 (0.138)<br>3.097 (0.008)	0.016
**20**	6.3 (0.3)	1.986 (0.004)	5.1 (0.6)	3.095 (0.009)	0.024
**25**	6.2 (0.3)	1.986 (0.005)	4.7 (0.7)	3.090 (0.010)	0.028
**30**	4.4 (0.5)<br>1.5 (0.5)	1.857 (0.013)<br>2.012 (0.014)	4. 7 (0.8)<br>2.0 (0.7)	2.891 (0.025)<br>3.0872 (0.036)	0.023

E_0=14 eV, $S_O^2=0.78$, $\sigma_{M-O}^2=0.007$, $\sigma_{M-M}^2=0.008$

Table S7. Location of XANES *K*-edge locations for $Co_{1-x}Fe_x(OH)_2$ sample series.[a]

	Water Series		Formamide Series	
**Fe (%)**	**Co K-edge**	**Fe K-edge**	**Co K-edge**	**Fe K-edge**
**0**	7720.90 (0.01)		7718.19 (0.01)	
**5**	7719.89 (0.04)	7124.21 (0.03)	7717.93 (0.09)	7124.58 (0.05)
**10**	7719.33 (0.03)	7124.22 (0.05)	7718.08 (0.08)	7124.55 (0.02)
**15**	7719.25 (0.20)	7124.21 (0.05)	7718.09 (0.09)	7124.36 (0.03)
**20**	7718.12 (0.12)	7124.14 (0.03)	7718.03 (0.07)	7124.03 (0.02)
**25**	7718.12 (0.13)	7124.08 (0.03)	7718.03 (0.02)	7123.78 (0.01)
**30**	7718.11 (0.01)	7123.82 (0.01)	7718.07 (0.01)	7123.60 (0.01)

[a] Standard deviation of all individual XANES scans taken as the estimated error

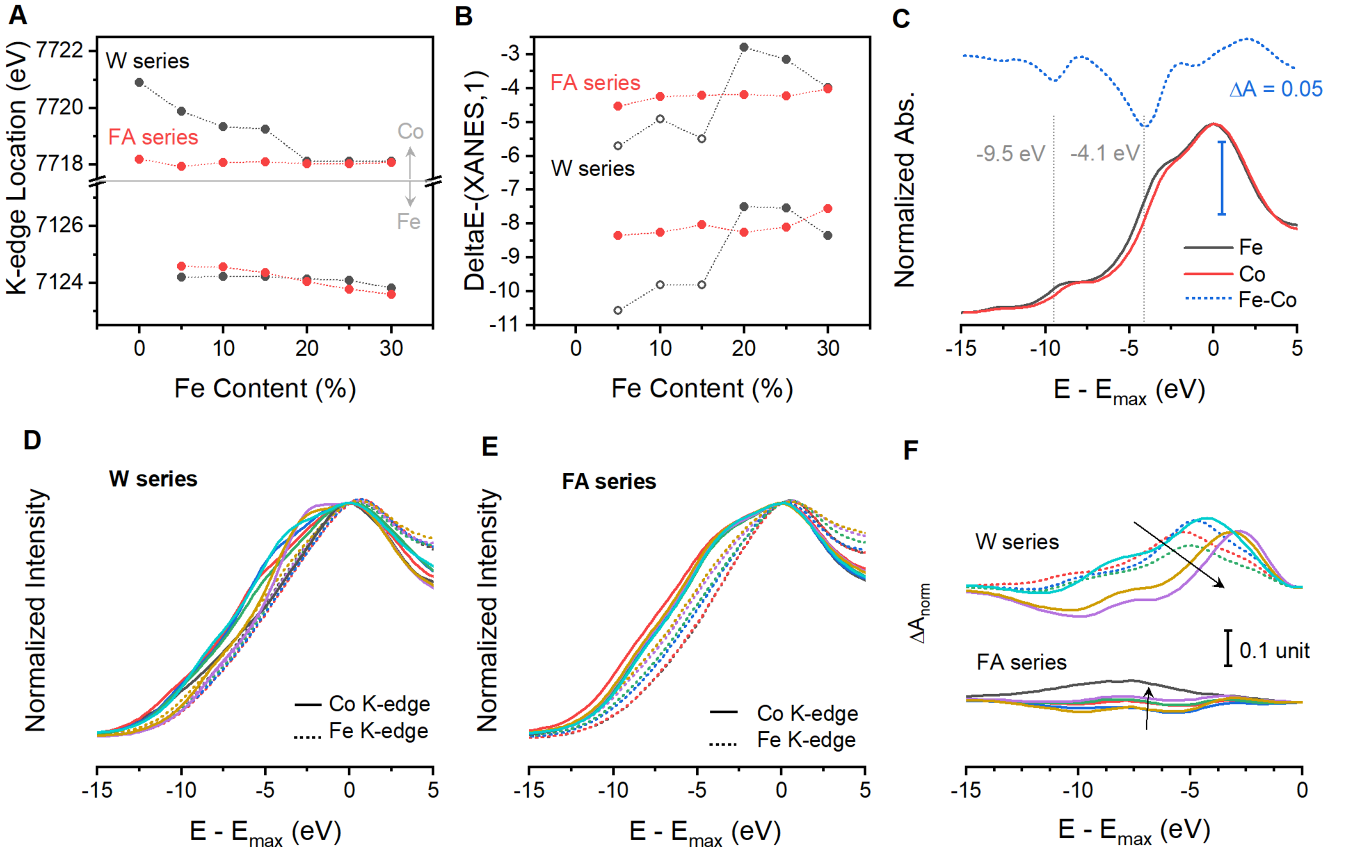

Figure S6. X-ray absorption near-edge spectrum analysis for $Co_{1-x}Fe_x(OH)_2$ composition series. **(A)** Location of Co (top) and Fe (bottom) *K*-edges as determined by the half-height method. **(B)** Location of XANES difference peaks as a function of composition. **(C)** XANES spectra calculated for $Co(OH)_2$ and $Fe(OH)_2$ with identical unit cells, the difference between the two plotted above. Overlay of all **(D)** water series and **(E)** formamide series spectra after normalizing and shifting the white line maxima. **(F)** XANES difference plots comparing the Co *K*-edge for samples containing Fe to the Co *K*-edge spectrum within the respective sample series.

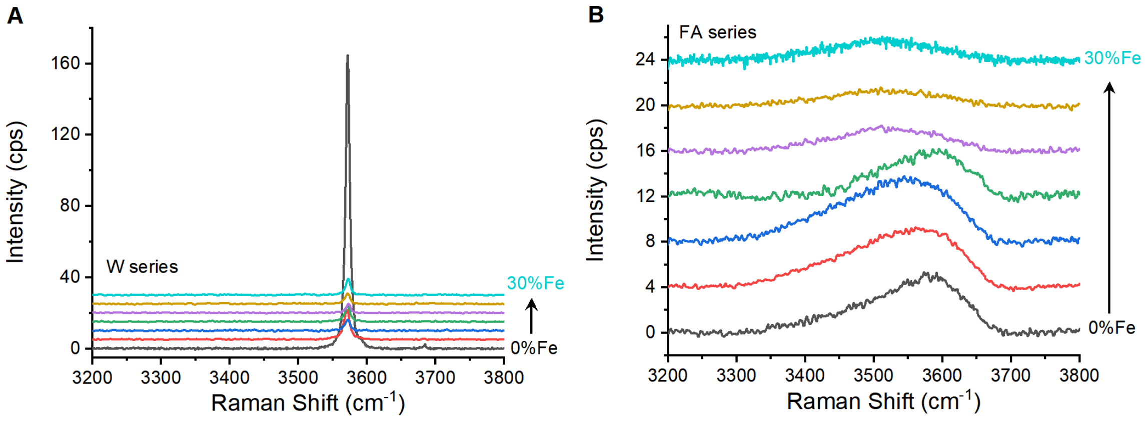

Figure S7. Raman spectra for $Co_{1-x}Fe_x(OH)_2$ in the high frequency region. Data shown for **(A)** water series and **(B)** the formamide series.

(A)

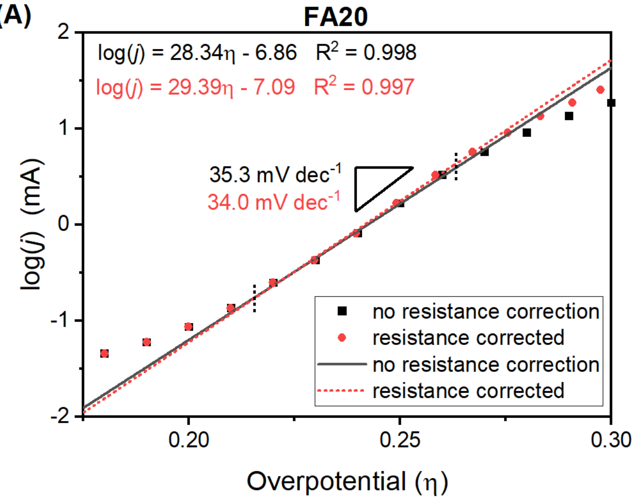

Equation	y = a + b*x	
Plot	logj	logj
Weight	No Weighting	
Intercept	-6.86451 ± 0.2026	-7.09787 ± 0.261
Slope	28.33712 ± 0.842	29.3883 ± 1.0898
Residual Sum of Squar	0.00213	0.00331
Pearson's r	0.99868	0.99794
R-Square (COD)	0.99735	0.99589
Adj. R-Square	0.99647	0.99452

(B)

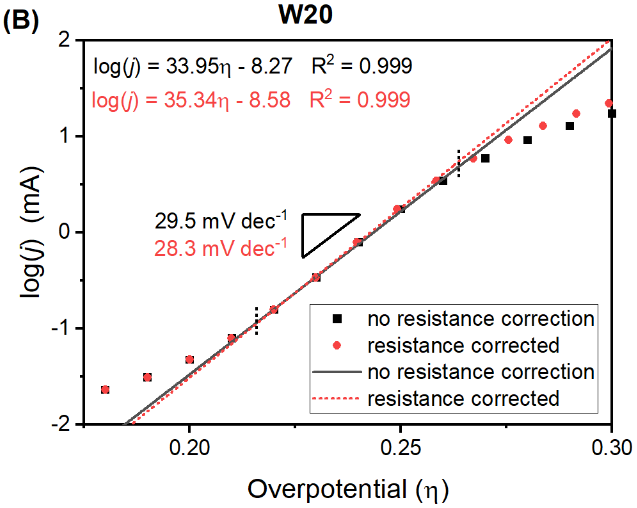

Equation	y = a + b*x	
Plot	logj	logj
Weight	No Weighting	
Intercept	-8.26908 ± 0.1639	-8.57969 ± 0.1123
Slope	33.95127 ± 0.682	35.33882 ± 0.468
Residual Sum of Squar	0.0014	6.07899E-4
Pearson's r	0.9994	0.99974
R-Square (COD)	0.99879	0.99947
Adj. R-Square	0.99839	0.9993

Figure S8. Demonstration of Tafel slope extraction from steady state chronoamperometric data. Linear regression analysis was performed on the strictly linear segment of the data, which spanned 5 to 7 data points. Comparison is shown for **(A)** FA20 and **(B)** W20 samples that were analyzed as-recorded, and with resistance correction by application of Ohm's law. Negligible differences between the two approaches led us to analyze the data without resistance correction. Short vertical dashed lines denote the linear region of the data from which the Tafel slope was extracted, and trend lines, linear fit equations, and noted Tafel slopes are colored for the as-recorded (black) and resistance corrected (red) data.

Table S8. Calculated bond angles and measured Tafel slopes used for correlational analysis.

	O-M-O Bond Angle (°)			Tafel Slope ($mV\ dec^{-1}$)	
**Sample**	**Co**	**Fe**	**Co - Fe**	**Average**	**St. Dev.**
**W0**	82.5	82.5	0.0	55.6	7.2
**W5**	90.8	74.0	16.9	35.2	4.0
**W10**	90.6	73.6	17.0	30.6	0.2
**W15**	90.2	73.8	16.3	29.4	1.1
**W20**	76.7	82.7	-6.0	30.8	1.4
**W25**	77.0	82.6	-5.6	31.4	3.0
**W30**	79.6	86.3	-6.7	29.3	2.0
**FA0**	81.9	81.9	0.0	42.4	3.5
**FA5**	79.7	80.3	-0.6	39.8	3.5
**FA10**	79.0	81.1	-2.1	37.5	3.7
**FA15**	79.0	82.2	-3.3	37.7	5.4
**FA20**	78.9	82.8	-3.9	36.3	1.1
**FA25**	79.3	84.1	-4.7	33.9	3.6
**FA30**	78.6	84.1	-5.5	35.9	3.0

References

(1) Anantharaj, S.; Noda, S.; Driess, M.; Menezes, P. W. The Pitfalls of Using Potentiodynamic Polarization Curves for Tafel Analysis in Electrocatalytic Water Splitting. *ACS Energy Lett.* **2021**, 1607–1611. https://doi.org/10.1021/acsenergylett.1c00608.

(2) Kibsgaard, J.; Chorkendorff, I. Considerations for the Scaling-up of Water Splitting Catalysts. *Nat. Energy* **2019**, *4* (6), 430–433. https://doi.org/10.1038/s41560-019-0407-1.

(3) Lazaridis, T.; Stühmeier, B. M.; Gasteiger, H. A.; El-Sayed, H. A. Capabilities and Limitations of Rotating Disk Electrodes versus Membrane Electrode Assemblies in the Investigation of Electrocatalysts. *Nat. Catal.* **2022**, *5* (5), 363–373. https://doi.org/10.1038/s41929-022-00776-5.

(4) Ehelebe, K.; Escalera-López, D.; Cherevko, S. Limitations of Aqueous Model Systems in the Stability Assessment of Electrocatalysts for Oxygen Reactions in Fuel Cell and Electrolyzers. *Current Opinion in Electrochemistry* **2021**, *29*, 100832. https://doi.org/10.1016/j.coelec.2021.100832.

(5) Haase, F. T.; Bergmann, A.; Jones, T. E.; Timoshenko, J.; Herzog, A.; Jeon, H. S.; Rettenmaier, C.; Cuenya, B. R. Size Effects and Active State Formation of Cobalt Oxide Nanoparticles during the Oxygen Evolution Reaction. *Nat. Energy* **2022**, *7* (8), 765–773. https://doi.org/10.1038/s41560-022-01083-w.
(6) Zheng, Y.-R.; Vernieres, J.; Wang, Z.; Zhang, K.; Hochfilzer, D.; Krempl, K.; Liao, T.-W.; Presel, F.; Altantzis, T.; Fatermans, J.; Scott, S. B.; Secher, N. M.; Moon, C.; Liu, P.; Bals, S.; Van Aert, S.; Cao, A.; Anand, M.; Nørskov, J. K.; Kibsgaard, J.; Chorkendorff, I. Monitoring Oxygen Production on Mass-Selected Iridium–Tantalum Oxide Electrocatalysts. *Nat. Energy* **2021**, *7* (1), 55–64. https://doi.org/10.1038/s41560-021-00948-w.
(7) Cherevko, S.; Geiger, S.; Kasian, O.; Kulyk, N.; Grote, J.-P.; Savan, A.; Shrestha, B. R.; Merzlikin, S.; Breitbach, B.; Ludwig, A.; Mayrhofer, K. J. J. Oxygen and Hydrogen Evolution Reactions on Ru, RuO 2 , Ir, and IrO 2 Thin Film Electrodes in Acidic and Alkaline Electrolytes: A Comparative Study on Activity and Stability. *Catalysis Today* **2016**, *262*, 170–180. https://doi.org/10.1016/j.cattod.2015.08.014.
(8) Guidelli, R.; Compton, R. G.; Feliu, J. M.; Gileadi, E.; Lipkowski, J.; Schmickler, W.; Trasatti, S. Defining the Transfer Coefficient in Electrochemistry: An Assessment (IUPAC Technical Report). *Pure Appl. Chem.* **2014**, *86* (2), 245–258. https://doi.org/10.1515/pac-2014-5026.
(9) Henstridge, M. C.; Laborda, E.; Compton, R. G. Asymmetric Marcus–Hush Model of Electron Transfer Kinetics: Application to the Voltammetry of Surface-Bound Redox Systems. *J. Electroanal. Chem.* **2012**, *674*, 90–96. https://doi.org/10.1016/j.jelechem.2012.04.006.
(10) Henstridge, M. C.; Laborda, E.; Wang, Y.; Suwatchara, D.; Rees, N.; Molina, Á.; Martínez-Ortiz, F.; Compton, R. G. Giving Physical Insight into the Butler–Volmer Model of Electrode Kinetics: Application of Asymmetric Marcus–Hush Theory to the Study of the Electroreductions of 2-Methyl-2-Nitropropane, Cyclooctatetraene and Europium(III) on Mercury Microelectrodes. *J. Electroanal. Chem.* **2012**, *672*, 45–52. https://doi.org/10.1016/j.jelechem.2012.02.028.
(11) Laborda, E.; Henstridge, M. C.; Compton, R. G. Asymmetric Marcus Theory: Application to Electrode Kinetics. *J. Electroanal. Chem.* **2012**, *667*, 48–53. https://doi.org/10.1016/j.jelechem.2011.12.011.
(12) Shinagawa, T.; Garcia-Esparza, A. T.; Takanabe, K. Insight on Tafel Slopes from a Microkinetic Analysis of Aqueous Electrocatalysis for Energy Conversion. *Sci Rep* **2015**, *5* (1), 13801. https://doi.org/10.1038/srep13801.
(13) Bockris, J. O. Kinetics of Activation Controlled Consecutive Electrochemical Reactions: Anodic Evolution of Oxygen. *J. Chem. Phys.* **1956**, *24* (4), 817–827. https://doi.org/10.1063/1.1742616.
(14) Bockris, J. O.; Otagawa, T. Mechanism of Oxygen Evolution on Perovskites. *J. Phys. Chem.* **1983**, *87* (15), 2960–2971. https://doi.org/10.1021/j100238a048.
(15) Lefebvre, M. C. Establishing the Link Between Multistep Electrochemical Reaction Mechanisms and Experimental Tafel Slopes. In *Modern Aspects of Electrochemistry*; Conway, B. E., Bockris, J. O., White, R. E., Eds.; Modern Aspects of Electrochemistry; Kluwer Academic Publishers: Boston, 2002; Vol. 32, pp 249–300. https://doi.org/10.1007/0-306-46916-2_3.
(16) Fabbri, E.; Schmidt, T. J. Oxygen Evolution Reaction—The Enigma in Water Electrolysis. *ACS Catal.* **2018**, *8* (10), 9765–9774. https://doi.org/10.1021/acscatal.8b02712.
(17) Sepa, D. B.; Vojnovic, M. V.; Stojanovic, M.; Damjanovic, A. Unconventional Symmetry Factor and Transfer Coefficient for Oxygen Reduction at Ir Electrodes and Their Possible

Use as Diagnostic Criteria in Mechanistic Analysis. *J. Electrochem. Soc.* **1987**, *134* (4), 845–848. https://doi.org/10.1149/1.2100584.
(18) Kötz, E. R.; Stucki, S. Ozone and Oxygen Evolution on PbO_2 Electrodes in Acid Solution. *J Electroanal. Chem. Interf. Electrochem.* **1987**, *228* (1–2), 407–415. https://doi.org/10.1016/0022-0728(87)80120-1.
(19) Kwabena Bediako, D.; Surendranath, Y.; Nocera, D. G. Mechanistic Studies of the Oxygen Evolution Reaction Mediated by a Nickel−Borate Thin Film Electrocatalyst. *J. Am. Chem. Soc* **2013**, *135*, 3662–3674. https://doi.org/10.1021/ja3126432.
